# A Pan-Cancer Analysis of Transcriptome Changes Associated with Somatic Mutations in *U2AF1* Reveals Commonly Altered Splicing Events

**DOI:** 10.1371/journal.pone.0087361

**Published:** 2014-01-31

**Authors:** Angela N. Brooks, Peter S. Choi, Luc de Waal, Tanaz Sharifnia, Marcin Imielinski, Gordon Saksena, Chandra Sekhar Pedamallu, Andrey Sivachenko, Mara Rosenberg, Juliann Chmielecki, Michael S. Lawrence, David S. DeLuca, Gad Getz, Matthew Meyerson

**Affiliations:** 1 Cancer Program, Broad Institute of Harvard and Massachusetts Institute of Technology, Cambridge, Massachusetts, United States of America; 2 Department of Medical Oncology, Dana-Farber Cancer Institute, Boston, Massachusetts, United States of America; 3 Department of Pathology, Harvard Medical School, Boston, Massachusetts, United States of America; Tel Aviv University, Israel

## Abstract

Although recurrent somatic mutations in the splicing factor *U2AF1* (also known as *U2AF35*) have been identified in multiple cancer types, the effects of these mutations on the cancer transcriptome have yet to be fully elucidated. Here, we identified splicing alterations associated with *U2AF1* mutations across distinct cancers using DNA and RNA sequencing data from The Cancer Genome Atlas (TCGA). Using RNA-Seq data from 182 lung adenocarcinomas and 167 acute myeloid leukemias (AML), in which *U2AF1* is somatically mutated in 3–4% of cases, we identified 131 and 369 splicing alterations, respectively, that were significantly associated with *U2AF1* mutation. Of these, 30 splicing alterations were statistically significant in both lung adenocarcinoma and AML, including three genes in the Cancer Gene Census, *CTNNB1*, *CHCHD7*, and *PICALM*. Cell line experiments expressing *U2AF1* S34F in HeLa cells and in 293T cells provide further support that these altered splicing events are caused by *U2AF1* mutation. Consistent with the function of U2AF1 in 3′ splice site recognition, we found that S34F/Y mutations cause preferences for CAG over UAG 3′ splice site sequences. This report demonstrates consistent effects of *U2AF1* mutation on splicing in distinct cancer cell types.

## Introduction

Recent whole-exome sequencing studies have identified recurrent somatic mutations in splicing factors in myelodysplastic syndromes [1], chronic lymphocytic leukemia [2], acute myeloid leukemia [3], breast cancer [4], lung adenocarcinoma [5], and uveal melanoma [6]. Many of these mutated splicing factors are branch point or 3′ splice site recognition factors (e.g., *SF3B1* and *U2AF1*) and are involved in regulating intron removal from pre-mRNA. The 3′ splice site recognition complex is composed of U2AF1 and U2AF2 (also known as U2AF65), where U2AF1 is important for the recognition of the AG at 3′ splice sites [7] and U2AF2 recognizes the polypyrimidine tract upstream of the AG [8].

*U2AF1* has been reported to have significant hotspot mutations at amino acid position 34 in myelodysplastic syndromes [1], lung adenocarcinomas [5], and AML [3]. *U2AF1* mutations co-occur with mutations in known driver oncogenes in lung adenocarcinoma [5] and it is yet unclear if these mutations are oncogenically transforming. To further elucidate core effects of *U2AF1* mutations on cancer biology, we aimed to identify common transcriptome alterations associated with *U2AF1* mutations in distinct cancer types.

## Results

### Somatic mutations in *U2AF1* across 12 cancer types

**Figure 1:**
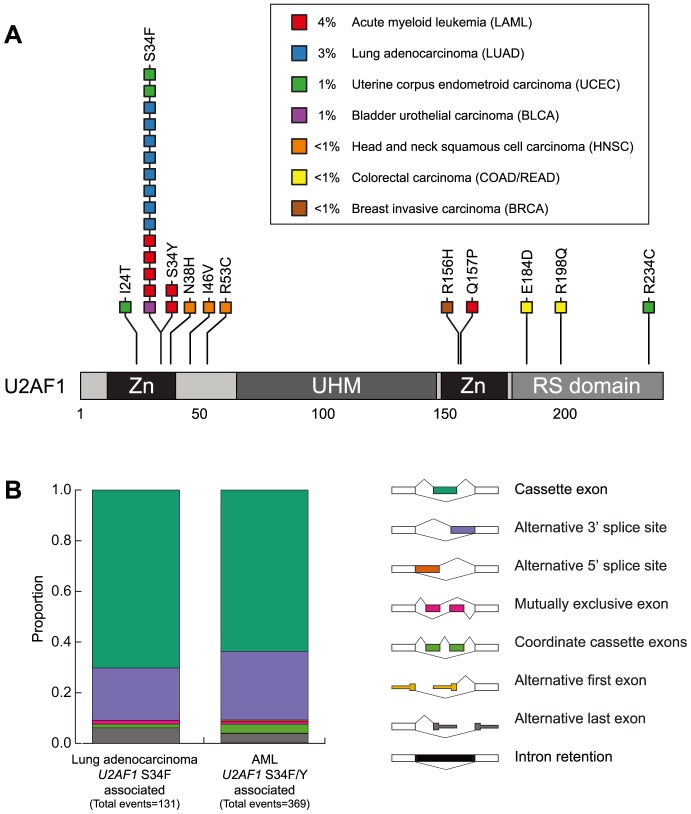
Splicing changes associated with *U2AF1* S34F/Y mutation in lung adenocarcinoma and AML. (**A**) Representation of somatic mutations in *U2AF1* observed across 12 TCGA cancer types [9]. Five cancer types had no somatic mutations in *U2AF1*. Amino acid positions are indicated. Zn, zinc finger; UHM, U2AF homology motif. (**B**) JuncBASE analysis of RNA-Seq data identified 131 and 369 splicing events significantly differentially spliced in lung adenocarcinoma and AML specimens bearing *U2AF1* S34F/Y mutations, respectively.

To look at transcriptome changes associated with *U2AF1* mutations in different cancer types, we used The Cancer Genome Atlas (TCGA) data that provide both somatic mutations at the DNA level and high-throughput sequencing of mRNA (RNA-Seq) in the same individual specimens across multiple cancer types. Using available data from 12 TCGA cancer types [9], we identified cancer harboring somatic mutations in *U2AF1*. Across 2,979 cancer specimens with both whole-exome sequencing and RNA-Seq, 26 had missense mutations in *U2AF1*—17 at amino acid position 34 (Table S1 in Document S1, Figure 1). *U2AF1* S34F mutations were found in eight lung adenocarcinomas (4%), four AML samples (2%), two endometrial carcinomas (1%), and one bladder cancer sample (1%). There were also two *U2AF1* S34Y AML samples (1%). *U2AF1* was reported to be significantly mutated in both lung adenocarcinomas [5] and AML [3]; however, not significantly mutated in endometrial carcinomas [10], most likely due to the low frequency within this cancer type. In addition to S34F/Y mutations, eight other somatic mutations were observed in *U2AF1*. To identify transcriptome alterations associated with *U2AF1* mutation, we focused on lung adenocarcinoma and AML as these cancer types have a higher frequency of mutations and thus would have more power to detect statistically significant changes associated with the mutation and control for differences between tissue of origin. The S34F and S34Y mutations result in aromatic amino acids; therefore, we consider S34F and S34Y mutations to be functionally similar.

### Transcriptome alterations associated with *U2AF1* S34F/Y mutations in lung adenocarcinoma and acute myeloid leukemia

We used JuncBASE [11] to identify alternative splicing events that were significantly differentially spliced between *U2AF1* wild-type cancer samples and *U2AF1* S34F/Y samples. JuncBASE identifies and classifies forms of alternative splicing (e.g., cassette exon, alternative 5′ splice sites, alternative 3′ splice sites), quantifies the abundance of the inclusion or exclusion of each alternative exon, then calculates a “percent spliced in” (PSI) value for each splicing event in each cancer sample. A Wilcoxon rank-sum test was performed on PSI values between cancers with or without *U2AF1* S34F/Y mutations to identify splicing events that were significantly different between the two groups. To control for tissue of origin, we compared *U2AF1* wild-type and S34F/Y samples within the same cancer type. The *U2AF1* S34F lung adenocarcinomas were found across the three expression subtypes (TCGA, *submitted*) and the *U2AF1* S34F/Y AML specimens occurred across five French-America-British (FAB) AML subtypes [3]; therefore, we did not further control for subtype. Somatic mutations in *U2AF1* have been found to be mutually exclusive with other splicing factors [1], [3], [5], suggesting that spliceosome pathway mutations may have the same functional effect on oncogenesis. To remove potential confounders from mutations in other splicing factors, we looked for differentially spliced events between samples with *U2AF1* S34F/Y mutation and samples harboring no mutation in any splicing factor gene that has been previously reported to be significantly altered in cancer (Tables S1–S2 in Document S1).

Using JuncBASE, we identified 131 and 369 alternative splicing events that were significantly differentially spliced in the presence of a *U2AF1* S34F/Y mutation in lung adenocarcinoma and AML (False Discovery Rate (FDR)<5%), respectively. Additionally, these splicing events have a >10% difference in the median PSI of cancers with no splicing factor gene mutation and cancers with *U2AF1* S34F/Y mutations (File S1–S3). Comparing *U2AF1* S34F/Y mutation with *U2AF1* wild-type samples (some with mutations in other splicing factors) yielded similar results in lung adenocarcinoma (TCGA, *submitted*) and AML (File S4). JuncBASE quantification of *U2AF1* S34F/Y-associated splicing events in AML were consistent with an analysis of *U2AF1* mutations in 20 AML samples [12](r^2^ = 0.84, Figure S1 in Document S1).

As both a positive and negative control for this analysis, we compared 10 lung adenocarcinoma samples with a *MET* exon 14 splice site mutation or deletion with all other lung adenocarcinoma samples to look for significant differential splicing between the two groups. The *MET* exon 14 alterations occur at a similar frequency as *U2AF1* mutations in lung adenocarcinoma (TCGA, *submitted*). This analysis serves as a positive control, because the most strongly associated differential splicing event should be *MET* exon 14. This analysis also serves as a negative control as the alterations would not be expected to cause global changes in splicing since the mutations occur at *cis*-acting splice site alterations. Indeed, JuncBASE analysis only identified the splicing of *MET* exon 14 as significantly differentially spliced with a difference in PSI >10%.

As an additional control, we identified alternative splicing events significantly associated with *STAG2* mutations in lung adenocarcinoma and AML, as *STAG2* is not expected to be a splicing regulator and it is mutated at a similar frequency in both cancer types. Only one splicing event was significantly associated with *STAG2* mutation in lung adenocarcinoma and none in AML.

Similar to what was observed in a comprehensive characterization of lung adenocarcinoma (TCGA, *submitted*), the splicing events associated with *U2AF1* S34F mutations in AML also show enrichment of cassette exons and alternative 3′ splice sites (Figure 1B). We do not observe broad effects on intron retention, which is thought to occur in the presence of a spliceosome mutation. If *U2AF1* mutations cause many introns to be unspliced, perhaps it is not in a consistent manner and those instances are cleared away by nonsense-mediated decay (NMD). To see if the cassette exon and 3′ splice site bias is a general feature of alternative splicing in these cancer types or specific to splicing altered by *U2AF1* mutation, we identified the proportions of each type of alternative splicing event in the top 10% most highly variable splicing events in lung adenocarcinomas without splicing factor mutations (Figure S2 in Document S1). *U2AF1* S34F/Y cancers preferentially exhibit alterations in cassette exon and 3′ splice sites compared to the set of highly variable splicing events (chi-squared test, P = 6.6e-26).

**Figure 2:**
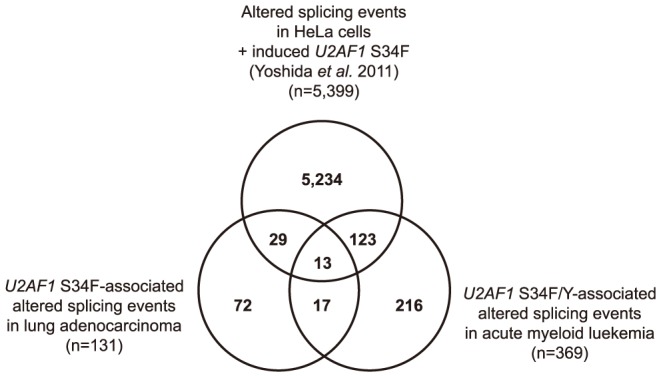
Commonly altered splicing events associated with *U2AF1* mutation in lung adenocarcinoma and AML. A comparison of changes in splicing associated with *U2AF1* somatic mutation in human cancers and *U2AF1* S34F induction in HeLa cells.

Although there are splicing changes that are associated with *U2AF1* S34F/Y mutation in these cancer specimens, it is unclear if the splicing changes are caused by the mutation or if there are unknown genomic alterations that are also correlating with the splicing changes. To evaluate further the relationship between the splicing changes and *U2AF1* S34F mutations, we analyzed previously published RNA-Seq data comparing ectopic expression of *U2AF1* S34F or *U2AF1* wild-type expression in HeLa cells [1]. JuncBASE analysis comparing no-induction controls with *U2AF1* wild-type or *U2AF1* S34F induction identified 1,221 and 5,399 significant splicing changes, respectively (File S5 and S6). We find a significant overlap of 165 splicing changes upon induction of *U2AF1* S34F in HeLa cells and splicing changes in human cancers with *U2AF1* S34F/Y mutations (Figure 2, Fisher's exact test, P<2.2e-16). In contrast, we find a smaller proportion of splicing changes upon *U2AF1* wild-type induction to be similar to alterations in human cancers (15/1221, Fisher's exact test, P = 0.72). In total, 35% of the *U2AF1* S34F/Y-associated splicing changes seen in lung adenocarcinoma or AML are supported by splicing changes caused by *U2AF1* S34F expression in HeLa cells. We would not expect all splicing changes to be observed in the HeLa cell experiments as splicing changes are known to be context dependent (reviewed in [13]) and *U2AF1* induction causes overexpression of the gene [1], which may affect spliceosome complex formation.

### *U2AF1* S34F/Y preferentially splices to CAG rather than UAG 3′ splice site sequences

U2AF1 is the small subunit of the U2AF heterodimer that recognizes the AG consensus of 3′ splice sites. A previous study that identified differential splicing of 35 exons associated with *U2AF1* mutation identified a sequence signature at 3′ splice sites of the alternative exons [12]. Given the significant changes in cassette exons and alternative 3′ splice site events associated with *U2AF1* S34F/Y mutation, we examined the sequences of 3′ splice sites that were preferentially used in *U2AF1* S34F/Y mutated cancers. For simplicity, we only examined splicing changes between two alternate 3′ splice sites.

**Figure 3:**
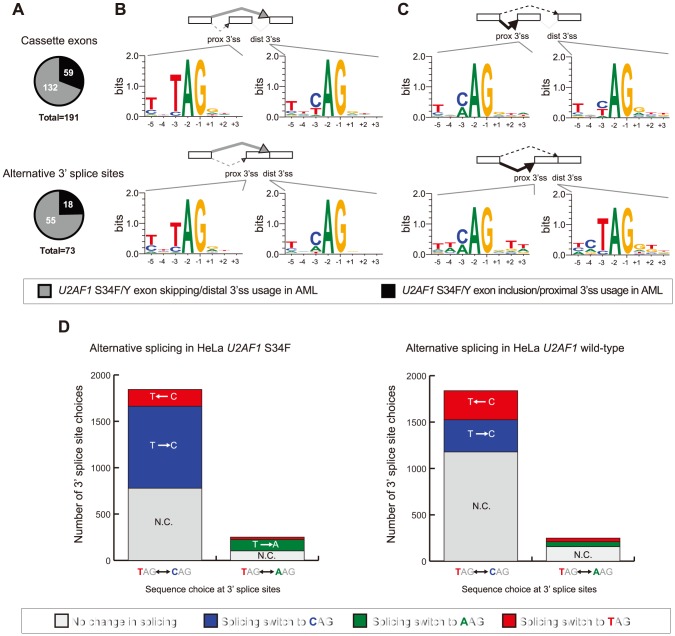
Sequence preferences at altered 3′ splice sites associated with *U2AF1* S34F/Y mutation. (**A**) Proportion of altered cassette exon and alternative 3′ splice site events that show exon skipping versus exon inclusion. (**B**) Consensus sequence motifs identified at the proximal (prox 3′ss) and distal 3′ splice sites (dist 3′ss) of exon skipping events. (**C**) Consensus sequence motifs at the proximal and distal 3′ splice sites of exon inclusion events. Splicing changes of expressed and annotated 3′ splice site choices where the splice site choice is TAG vs. CAG or TAG vs. AAG in (**D**) HeLa cells+induced *U2AF1* S34F or (**E**) HeLa cells+induced *U2AF1* wild-type.

As splicing factor binding can differ depending on splicing activation or repression (reviewed in [14]), we looked at splice site sequences of exon inclusion and exclusion events, separately. From the set of *U2AF1* S34F/Y-associated cassette exons and alternative 3′ splice site changes in AML, we found a significant bias toward skipping of an exon (69% of cassette exons) or usage of a more distal 3′ splice site (75% of alternative 3′ splice sites) (binomial test, P<1e-4, Figure 3A). We found a similar bias toward exon skipping and distal splice site usage of *U2AF1* S34F-associated splicing events in lung adenocarcinoma (Figure S3 in Document S1). As a comparison, we identified alternative splicing events significantly associated with *RBM10* mutation in lung adenocarcinoma (File S7). The RNA binding protein *RBM10* was found to be significantly mutated in lung adenocarcinomas with recurring frameshift, nonsense, or splice site mutations [5]. The observed exon skipping is specific to *U2AF1* S34F-associated splicing events as the opposite bias is observed in *RBM10* loss-of-function (LOF)-associated splicing events (Figure S4A–B in Document S1).

Consistent with previous findings [12], we found a trinucleotide consensus TAG sequence at proximal 3′ splice sites of skipped cassette exons. Moreover, we also find this motif at proximal splice sites of alternative 3′ splice site events in *U2AF1* S34F/Y AML samples (Figure 3B). Additionally, a consensus CAG splice site at the more distal 3′ splice site is observed, which has not been previously reported (Figure 3B). A similar preference of CAG splice sites (with a minor AAG sequence) over TAG splice sites is observed in exons preferentially included in *U2AF1* S34F/Y AML samples (Figure 3C). These preferential splice site sequence motifs differ from splice site motifs observed near proximal and distal 3′ splice sites of control alternative splicing events (Figure S4C in Document S1, Wilcoxon rank-sum test, FDR >95%).

Strikingly, we see the same sequence preferences in cassette exon and alternative 3′ splice site changes in *U2AF1* S34F lung adenocarcinomas (Figure S3 in Document S1) and in HeLa cells transduced with *U2AF1* S34F mutant constructs (Figure S5A–B in Document S1). Moreover, we do not see this consensus 3′ splice site sequence in *RBM10* LOF-associated splicing in lung adenocarcinoma (Figure S4A–B in Document S1) nor in splicing changes in HeLa cells transduced with wild-type *U2AF1* (Figure S5C–D in Document S1), supporting that the sequence preference is specific to *U2AF1* S34F/Y mutations.

These consensus sequence motifs were generated from splicing events that were strongly associated with *U2AF1* mutation and do not resolve sequence preferences between two splice site choices from an individual splicing event. To further investigate the CAG splice site preferences over UAG splice sites, we examined splice site sequence switches between all pairs of expressed and annotated CAG versus TAG 3′ splice sites. Using a lowered threshold for identifying splicing switches, we found that 57% of CAG versus TAG 3′ splice sites were altered in HeLa cells expressing the *U2AF1* S34F mutation, while 36% were altered when expressing a *U2AF1* wild-type construct (Figure 3D). Of *U2AF1* S34F-associated splice site switches, 83% (884/1,065) were a TAG to a CAG splice site, while only 53% (347/660) switched from a TAG to a CAG when expressing U2AF1 wild-type (Fisher's exact test, P<2.2e-16). A similar bias of switches from TAG to AAG 3′ splice sites was observed (Fisher's exact test, P = 2.518e-5, Figure 3D).

Our analysis shows that the *U2AF1* S34F/Y mutation causes alterations in 3′ splice site usage where a [C/A]AG 3′ splice site is preferred over a UAG splice site. We did not observe altered splicing of all UAG 3′ splice sites; however, it is difficult to distinguish between unaltered splicing or degradation of the aberrant transcript by nonsense-mediated decay, thus appearing to be unchanged.

### Transcriptome alterations in cell cycle genes and other cancer genes are associated with *U2AF1* mutation

Somatic mutation in *U2AF1* may cause splicing changes of key cancer genes, resulting in altered gene function or altered pathways. To identify potential downstream biological consequences of *U2AF1* mutation, we performed a Gene Set Enrichment Analysis (GSEA) [15], [16] to identify gene sets positively correlated with the *U2AF1* S34F/Y mutation in lung adenocarcinoma and in AML. No gene sets reached statistical significance given an FDR cutoff of 25%. It was previously reported that transduction of *U2AF1* S34F mutations in HeLa cells caused upregulation of components of the nonsense-mediated decay (NMD) pathway [1]. We did not observe a significant upregulation in expression of NMD factors associated with *U2AF1* mutation in lung adenocarcinoma or AML.

Although no gene sets passed FDR significance, the top-two enriched GO ontology gene sets in AML with the highest normalized enrichment scores were “mitotitc cell cycle” and “M phase of mitotic cell cycle” (Figure S6 in Document S1, nominal P<0.05). A subset of these cell cycle genes were upregulated in AML samples with *U2AF1* S34F/Y mutation. Consistent with the effect of *U2AF1* mutation on the cell cycle, RNAi depletion of *U2AF1* [17] and *U2AF1* S34F expression in HeLa cells [1] results in an increased fraction of cells in G2/M phase. We do not see any association with cell cycle gene sets in *U2AF1* S34F lung adenocarcinomas. This may be explained by the high normal tissue contamination in this cancer type [18], which may affect gene expression signatures.

In line with these observations, there are 31 genes with *U2AF1* S34F/Y-associated splicing events represented in the “mitotic cell cycle” and “M phase of mitotic cell cycle” gene sets, including *CHEK2*, *CDC27*, and multiple subunits of the anaphase-promoting complex (Table S3 in Document S1).

Splicing alterations associated with *U2AF1* S34F/Y mutation in both lung adenocarcinoma and AML are of particular interest as these commonly altered genes may make core contributions to selective advantage in these cancers. We found a significant overlap of 30 altered splicing events that were associated with *U2AF1* S34F/Y mutations in both lung adenocarcinoma and AML (Figure 2 and Table 1, Fisher's exact test p<2.2e-16). For all 30 splicing events, the direction of splicing was the same in both cancer types, where *U2AF1* mutation caused a shift to exon skipping or exon inclusion for the same splicing event. The majority (63%) of the commonly altered events showed more distal 3′ splice site usage in *U2AF1* mutated samples. In addition, 17/30 splicing events were significantly affected upon induction of *U2AF1* S34F in HeLa cells but had no change upon induction of *U2AF1* wild-type (Table 1). 60% of these commonly altered splicing events show 3′ splice site sequence preference of a CAG, or AAG, splice site over a TAG splice site (Table 1, a or b), consistent with the overall observed splice site sequence preference of *U2AF1* S34F/Y-associated events. Additionally, we looked for genes with altered splicing events that were also in the Cancer Gene Census [19] (Table S4 in Document S1). Three Cancer Gene Census genes were among the 30 commonly altered splicing events and are of particular interest: *CTNNB1* (TCGA, *submitted*), *CHCHD7*, and *PICALM* (Table 1 and Table S4 in Document S1).

**Table 1:** 30 significant splicing alterations associated with *U2AF1* S34F/Y mutations in both lung adenocarcinoma and AML.

Gene	AS type	Distal 3′ splice site usage?	U2AF1 S34F HeLa?	Proximal splice site	Distal splice site	Strand	Splice site pref.
*ACAD8*	CE	Y	Y	chr11:134126981	chr11:134128408	+	a
*C12orf11*	CE	Y	Y	chr12:27067512	chr12:27067062	−	a
*C17orf45*	A3S	Y	Y	chr17:16342841	chr17:16342973;chr17:16342894	+	c
*CHCHD7*	A3S	Y	Y	chr8:57128947	chr8:57128991	+	b
*CTNNB1*	A3S		Y*	chr3:41281150	chr3:41281309	+	c
*FAM60A*	CE		Y	chr12:31458058	chr12:31451159	−	c
*FXR1*	CE		Y	chr3:180693100	chr3:180693909	+	c
*GUSB*	CE	Y	Y	chr7:65440059	chr7:65439692	−	c
*HMGCR*	CE	Y	Y	chr5:74650880	chr5:74651189	+	c
*KARS*	CE	Y	Y	chr16:75678361	chr16:75675622	−	a
*KIAA0182*	A3S	Y		chr16:85696949	chr16:85696991	+	a
*MAP3K3*	CE			chr17:61712068	chr17:61723393	+	c
*MARK3*	A3S	Y		chr14:103934369	chr14:103934417	+	b
*MR1*	CE	Y	Y	chr1:181021370	chr1:181022708	+	a
*NSUN2*	CE	Y		chr5:6632091	chr5:6625783	−	a
*PABPC4*	CE	Y		chr1:40029595	chr1:40029414	−	b
*PICALM*	A3S			chr11:85693047	chr11:85693032	−	b
*PPHLN1*	CE	Y	Y	chr12:42745686	chr12:42748962	+	a
*PPM1B*	ALE			chr2:44457551	chr2:44459454	+	b
*PTBP1*	CE	Y		chr19:805512	chr19:806407	+	a
*RIPK2*	CE	Y	Y	chr8:90775056	chr8:90777568	+	b
*SERF1B*	ALE	Y		chr5:69328141	chr5:69337350	+	a
*SERF1B*	ALE	Y		chr5:70203560	chr5:70212767	+	a
*SETD4*	CE		Y	chr21:37429776	chr21:37429503	−	b
*SETX*	CE		Y	chr9:135144877	chr9:135140373	−	c
*STRAP*	CE	Y		chr12:16036474	chr12:16042861	+	c
*TMEM131*	CE	Y	Y	chr2:98411579	chr2:98410017	−	a
*USP25*	CE			chr21:17222095	chr21:17236586	+	c
*USP33*	A3S		Y	chr1:78187611	chr1:78187587	−	a
*ZRANB2*	CE			chr1:71531436	chr1:71530821	−	c

CE, cassette exon; A3S, alternative 3′ splice site; ALE, alternative last exon;

* , *U2AF1* wild-type induction caused differential splicing, but in the opposite direction as *U2AF1* S34F induction; Splice site preference in U2AF1 S34F/Y samples: a, TAG→CAG; b, TAG→AAG; c, other.

**Figure 4:**
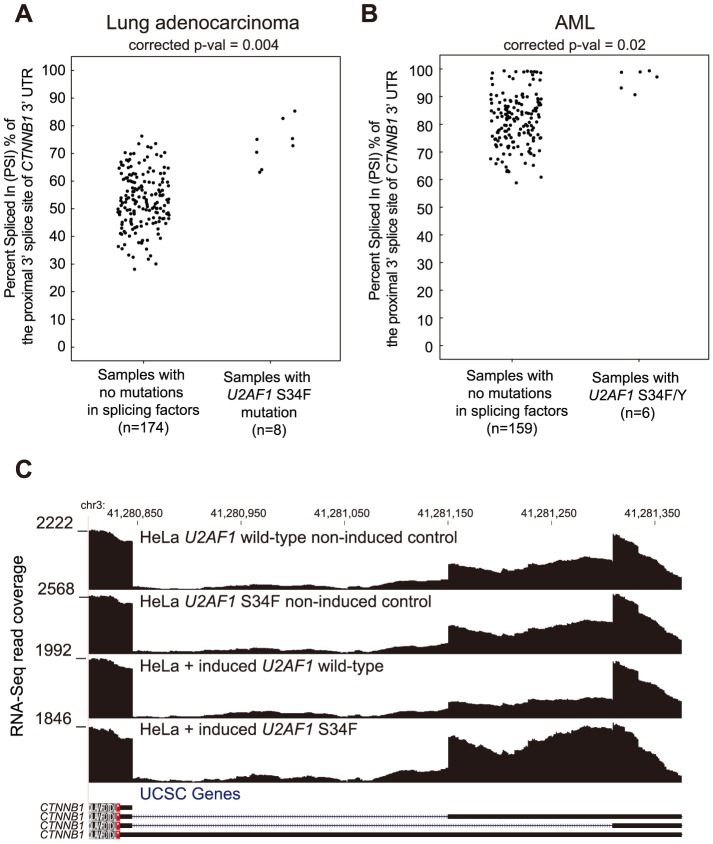
*CTNNB1* 3′ UTR splicing associated with *U2AF1* S34F/Y mutation in lung adenocarcinoma and AML. “Percent spliced in” (PSI) values of the proximal 3′ splice site of the *CTNNB1* 3′ UTR splice event in (**A**) lung adenocarcinoma and (**B**) AML. (**C**) RNA-Seq read coverage of the 3′ UTR event in HeLa cells with two *U2AF1* non-induced controls, induction of *U2AF1* wild-type, and induction of *U2AF1* S34F.

The altered splicing event in *CTNNB1* in *U2AF1* mutant cancers results in a shift toward splicing of a more proximal splice site in the 3′ UTR of *CTNNB1* (Figure 4A–B). Significant upregulation of proximal splice site usage was also observed in HeLa cells expressing *U2AF1* S34F (Figure 4C). The upregulated isoform has been shown to be a less stable mRNA in HeLa cells [20]; therefore, the altered splicing event may result in a reduction of CTNNB1 protein levels. Although, *CTNNB1* activation is a canonical driver mutation, a decrease in normal levels of CTNNB1 may also be advantageous to cancer cells as RNAi depletion of *CTNNB1* has been shown to increase cell migration [21].

**Figure 5:**
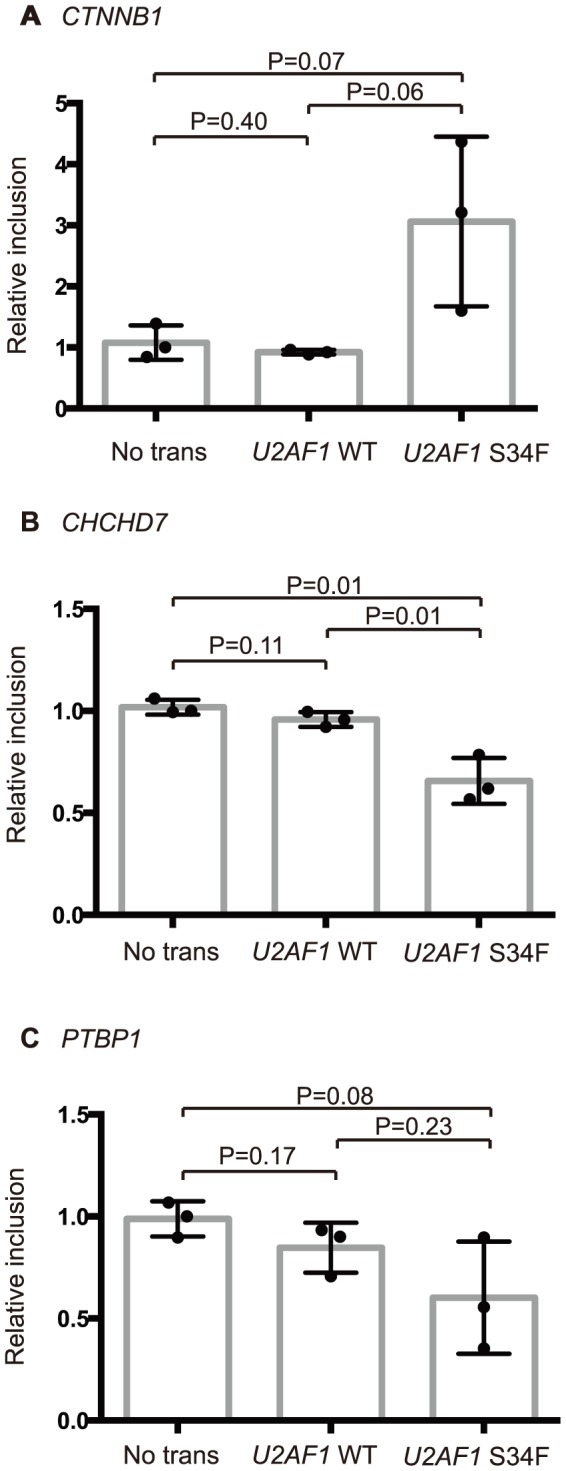
Quantitative RT-PCR validation of splicing events affected by expression of *U2AF1* S34F in 293T cells. A fold difference between total gene expression and the inclusion isoform of each splicing event was normalized by the fold difference of a no-transfection control sample to yield a relative inclusion level for each sample. The genomic coordinates of tested splice events in (**A**) *CTNNB1*, (**B**) *CHCHD7*, and (**C**) *PTBP1* are given in Table 1.

To further test experimentally whether the splicing events observed in *U2AF1* mutant cancers are directly caused by *U2AF1* mutation, we transfected 293T cells with either *U2AF1* wild-type or *U2AF1* S34F constructs and assayed changes in five splicing events using quantitative RT-PCR (Figure 5, Figure S7 in Document S1). We were able to validate changes in splicing in *CTNNB1*, *CHCHD7*, and *PTBP1* in cells transfected with *U2AF1* S34F constructs and not in cells transfected with *U2AF1* wild-type (Figure 5, unpaired t-test, P<0.1). Splicing events in *KARS* and *CHEK2* did not validate and may be context dependent, false-positives in the transcriptome analysis, false negatives in the transfection experiment, or indirect delayed targets of *U2AF1* activity.

## Discussion

Our analysis of splicing and gene expression changes associated with *U2AF1* mutation across multiple cancer types has revealed alterations in 3′ splice site sequence recognition and highlights potential biological consequences through altered cancer genes, such as *CTNNB1*, *PICALM*, and *CHCHD7*. In addition, we have validated that at least some of these splicing alterations are direct consequences of *U2AF1* mutation in transfection experiments.

Many factors are involved in correct splice site recognition in complex with U2AF1, including U2AF2. It has been well know that the 3′ splice site consensus motif includes a C or U nucleotide before the AG at the end of introns and mutations in *U2AF1* give the first indication that a factor has a specific preference for this −3 position [12]. U2AF1 has been shown to weakly bind to RNA via it's UHM domain [22]. Perhaps the S34F mutation in the upstream zinc finger domain affects 3′ splice site sequence recognition via direct interaction with the pre-mRNA or through protein-protein interactions with U2AF2, hnRNP A1 [23], or DEK [24]. Further biochemical characterization of the U2AF1 S34F protein in complex with other proteins may lead to a more general understanding of 3′ splice site sequence recognition.

In summary, we found multiple genes including several known cancer genes with altered splicing in the presence of the *U2AF1* mutation both in human cancer and in *U2AF1* mutant transfected cells. The impact of these splicing alterations on gene function is an important topic for future investigation. Our identification of splicing changes significantly and consistently associated with somatic mutations in the *U2AF1* splicing factor highlights the need for future study into the function of alternative splice forms of genes in order to fully understand the genomic alterations associated with cancer.

## Methods

### Somatic mutations

Somatic mutation calls from the 12 TCGA Pan-Cancer cancer types were used (doi:10.7303/syn1710680.4), with the exception of lung adenocarcinoma. Lung adenocarcinoma somatic mutation calls were taken from the TCGA lung adenocarcinoma analysis working group (TCGA, *submitted*).

### RNA-Seq processing

RNA-Seq alignment files for 230 lung adenocarcinomas and 173 acute myeloid leukemias (AML) were available through CGHub (https://cghub.ucsc.edu). AML alignments downloaded from CGHub used an older version of the human genome reference (hg18); therefore, the AML BAM files were converted to FASTQ using SamToFastq [25] and then realigned against hg19 using TopHat [26] with the following parameters: “-G [Gencode v7 transcripts [27]] –mate-inner-dist 300 –mate-std-dev 500”. One AML sample failed RNA-Seq re-alignment and processing and was excluded: TCGA-AB-2977. RNA-Seq alignments of lung adenocarcinoma samples used MapSplice [28].

RNA-Seq FASTQ files from HeLa cell experiments were downloaded from the DDBJ repository with the accession numbers DRR001796-DRR001799. Reads were aligned using TopHat [26] using Gencode v7 [27] as a transcript guide.

### Splicing analysis

Lung adenocarcinoma samples, AML samples, and samples from the HeLa cell line experiments (HeLa+*U2AF1* wild-type non-induced, HeLa+*U2AF1* S34F non-induced, HeLa+*U2AF1* wild-type induced, HeLa+*U2AF1* S34F induced) were run through JuncBASE v0.6 [11] as three separate analysis sets. For lung adenocarcinoma and AML samples, the JuncBASE parameters used to identify alternative splicing events and calculate “percent spliced in” (PSI) values were the following: *-c 2 -j [introns from Gencode v7* [27] *] –jcn_seq_len 88*. For the HeLa cell experiments, the JuncBASE parameters used were the following: *-c 2.5 -j [introns from Gencode v7* [27] *] –jcn_seq_len 202*. For lung adenocarcinoma, Cufflinks [29] *de novo* transcript annotations were available to incorporate potentially novel exons in the splicing analysis (TCGA, *submitted*). UCSC knownGenes hg19 transcript annotation was used to define annotated exons [30].

To identify *U2AF1* S34F/Y-associated differential splicing events in lung adenocarcinoma and AML, *compareSampleSets.py* of the JuncBASE v0.6 package was used with the following parameters: *–thresh 10 –mt_correction BH –which_test Wilcoxon –delta_thresh 5.0*. Splicing events with a significant difference (FDR<5%) in PSI values between samples with no spicing factor mutation and samples with *U2AF1* S34F/Y mutation were further filtered for events with a difference in the median PSI greater than 10%. To identify splicing events affected by expression of *U2AF1* S34F in HeLa cells, *pairwise_fishers_test_getASEvents_w_reference.py* was used to compare inclusion and exclusion isoform read counts between the HeLa+*U2AF1* wild-type non-induced sequenced library and HeLa+*U2AF1* S34F-induced sequenced library with the following parameters: *–thresh 25 –min_dpsi_threshold 5.0 –method BH –sign_cutoff 0.05*. To identify splicing events specific to *U2AF1* S34F induction, the event could not also be significantly different between the two controls or when comparing the *U2AF1* wild-type non-induced to *U2AF1* wild-type induced. This last restriction was imposed to distinguish splicing changes due to the S34F mutation instead of overexpression of *U2AF1*. In some instances, such as the 3′ UTR splicing event of *CTNNB1*, *U2AF1* wild-type induction caused a change in splicing but in the opposite direction as *U2AF1* S34F induction. To be conservative, we consider a *U2AF1* S34F-affected splicing event that has any change upon *U2AF1* wild-type induction to be non-specific to the *U2AF1* mutation; however, we make note in Table 1 of the *CTNNB1* event that also showed a splicing change in *U2AF1* wild-type induction but in the opposite direction.

In cases where there are more than two alternatives for a splicing event (e.g., three alternative 3′ splice site choices), the most significant differentially expressed isoform is reported.

To identify *U2AF1* S34F/Y-associated differential splicing in both lung adenocarcinoma and AML, we checked for overlap in the genomic coordinates of the splice event from each cancer type. We found 31 splicing events that occurred at the same genomic location of the genes; however, the event in *ASPH* was not the same splicing event upon manual review and was not included in Table 1. To test for the significance of the 30 commonly altered splicing events in lung adenocarcinoma and AML, we used a Fisher's exact test where the 2×2 table contained the number of splicing events affected and unaffected by the *U2AF1* S34F/Y mutation in the two cancer types. Only splicing events that were expressed in both cancer types were used in the 2×2 table.

The list of Cancer Gene Census [19] genes were downloaded on May 31, 2013 to compare against the list of *U2AF1* S34F/Y-associated splicing events.

### 3′ splice site consensus sequences

The splice site sequence analysis used *U2AF1* S34F/Y-associated differentially spliced cassette exons and alternative 3′ splice sites where only two alternate splice sites were observed from the RNA-Seq data. The direction of splicing (exon inclusion or skipping) was determined by taking the difference in the median PSI value between samples with no splicing factor mutation and samples with a *U2AF1* S34F/Y mutation. For example, if the median PSI was lower in the *U2AF1* mutated samples, the alternative exon was preferentially skipped in the presence of the mutation.

3′ splice site sequences from the proximal and distal splice sites of each splicing event were extracted from genomic coordinates by taking 20 nt upstream and 3 nt downstream of the intron/exon boundary. Splice site sequences were used as input to WebLogo 3 [31], [32] to generate consensus motif logos. Motif logos were only generated for cases with 10 or more exon inclusion or exclusion events.

### Sequence preference at 3′ splice sites

Cassette exon and alternative 3′ splice site events involving only annotated introns from Gencode v7 [27] were used to further examine 3′ splice site sequence preferences of U2AF1 S34F when expressed in HeLa cells. Splicing events that were detectable with at least 25 reads and had only two splice site choices were considered. To look more globally at splicing switches, we used a lower threshold of a change in PSI >5%, no statistical cutoff, and no consideration of inclusion or exclusion of an alternative region, as our criteria for calling a splicing switch. HeLa cells expressing U2AF1 S34F or U2AF1 wild-type were compared to their matched non-induced controls to determine if a splicing switch occurred.

### Gene Set Enrichment Analysis (GSEA)

Gene expression quantification from RNA-Seq data was taken from the TCGA lung adenocarcinoma study (TCGA, *submitted*) and from the TCGA Pan-Cancer Analysis Group for AML samples (doi:10.7303/syn1681084.1). Gene expression for both sets were given as RSEM values [33] and were pre-filtered before performing Gene Set Enrichment Analysis (GSEA) analysis [15], [16]. The gene expression values were filtered to remove unexpressed genes (RSEM values<0) and genes with low variance (fold change between the minimum and maximum value >1.5). The GO annotation database (c5.bp.v3.1.symbols.gmt) and the set of nonsense-mediated decay (NMD) factors listed in Yoshida et al. 2011 [1] were used as gene set inputs to GSEA. Significance was derived from 10,000 permutations of the phenotypes *U2AF1* S34F/Y vs wild-type.

### Data visualization

Plots comparing PSI values in samples with no splicing factor mutation to samples with *U2AF1* S34F/Y samples were created with the *ggplot2* (*ggplot2.org*) library in R [34]. RNA-Seq coverage plots were generated by converting BAM files to bigWig files using *samtools* [25], the *wiggles* script from TopHat [26], *wigToBigWig* [30], and visualized using the UCSC genome browser (http://genome.ucsc.edu) [30].

### Validation experiments in 293T cells

293T cells were transfected with V5 tagged *U2AF1* WT or *U2AF1* S34F constructs in biological triplicate and expression was confirmed by western blot (Figure S7 in Document S1). Quantitatitve RT-PCR was performed using Taqman Universal PCR Master Mix (Life Technologies) on cDNA from non-transfected and transfected samples. Taqman primers and probes for five splicing events were selected for constitutive regions of each gene to detect changes in total isoform expression and also selected for a splice junction specific to the inclusion isoform (Table S5 in Document S1). Custom probe sets were designed using the Custom TaqMan Assay Design Tool (Life Technologies) when necessary. Assays were run on the ABI 7300 Real-Time PCR System under standard conditions. The fold difference between the inclusion isoform and total gene expression was calculated for each sample as 2^−(Ct(inclusion)-Ct(total))^. Fold differences were than normalized by the median fold difference observed in non-transfected samples to get a relative inclusion level for each splicing event.

## Supporting Information

Document S1 **Supporting figures and tables.** **Figure S1**, Correlation of ΔPSI values between JuncBASE and Przychodzen *et al.* [1]. **Figure S2**, Highly variable alternative splicing events in lung adenocarcinoma. **Figure S3**, Splice site motifs at cassette exon and alternative 3′ splice site changes associated with *U2AF1* S34F mutation in lung adenocarcinoma. **Figure S4**, Splice site motifs at cassette exon and alternative 3′ splice site changes associated with *RBM10* loss-of-function (LOF) mutation in lung adenocarcinoma and control alternative splicing events. **Figure S5**, Splice site motifs at cassette exon and alternative 3′ splice site changes associated with induction of *U2AF1* S34F or *U2AF1* wild-type in HeLa cells. **Figure S6**, GSEA enrichment analysis in AML samples. **Figure S7**, Expression of V5 tagged U2AF1 WT or U2AF1 S34F constructs in 293T cells as determined by western blot. **Table S1**, The Cancer Genome Atlas sample identifiers used in this study. Somatic mutations in splicing factors are indicated. **Table S2**, Splicing factors reported to be significantly altered in previous studies where *U2AF1* is somatically mutated. **Table S3**, Mitotic cell cycle genes that show differential splicing in the presence of the *U2AF1* S34F/Y mutation. **Table S4**, Cancer Gene Census genes differentially spliced in the presence of a *U2AF1* S34F/Y mutation. **Table S5**, Taqman assays (Life Technologies) used for quantitative RT-PCR of splicing events in *CHCHD7*, *CHEK2*, *CTNNB1*, *KARS*, and, *PTBP1*.
(PDF)

File S1 Column description for Files S2, S3, S4, S5, S6, S7. Description of columns in Files S2, S3, S4, S5, S6, S7 describing alternative splicing events.
(XLSX)

File S2 *U2AF1* S34F-associated alternative splicing events in lung adenocarcinoma. *U2AF1* S34F specimens versus specimens with no splicing factor mutation. *U2AF1* S34F-associated alternative splicing events in lung adenocarcinoma when comparing specimens that are *U2AF1* wild-type was reported in a study of genome alterations in lung adenocarcinoma (TCGA, *submitted*).
(TXT)

File S3 *U2AF1* S34F/Y-associated alternative splicing events in AML, version 1. *U2AF1* S34F/Y specimens versus specimens with no splicing factor mutation.
(TXT)

File S4 *U2AF1* S34F/Y-associated alternative splicing events in AML, version 2. *U2AF1* S34F/Y specimens versus specimens that are *U2AF1* wild-type. Some wild-type specimens have mutations in other splicing factors.
(TXT)

File S5 Splicing changes upon induction of *U2AF1* wild-type in HeLa cells when compared to no-induction controls.
(TXT)

File S6 Splicing changes upon induction of *U2AF1* S34F in HeLa cells when compared to no-induction controls.
(TXT)

File S7 *RBM10 LOF*-associated alternative splicing events in lung adenocarcinoma. *RBM10* LOF specimens versus specimens with no splicing factor mutation.
(TXT)
