# Correction: A Pan-Cancer Analysis of Transcriptome Changes Associated with Somatic Mutations in *U2AF1* Reveals Commonly Altered Splicing Events

**DOI:** 10.1371/journal.pone.0096437

**Published:** 2014-04-29

**Authors:** The PLOS ONE Staff

The graph legend for Figure 3D does not display correctly. The publisher apologizes for this error. Please find a corrected version below.

**Figure 3:**
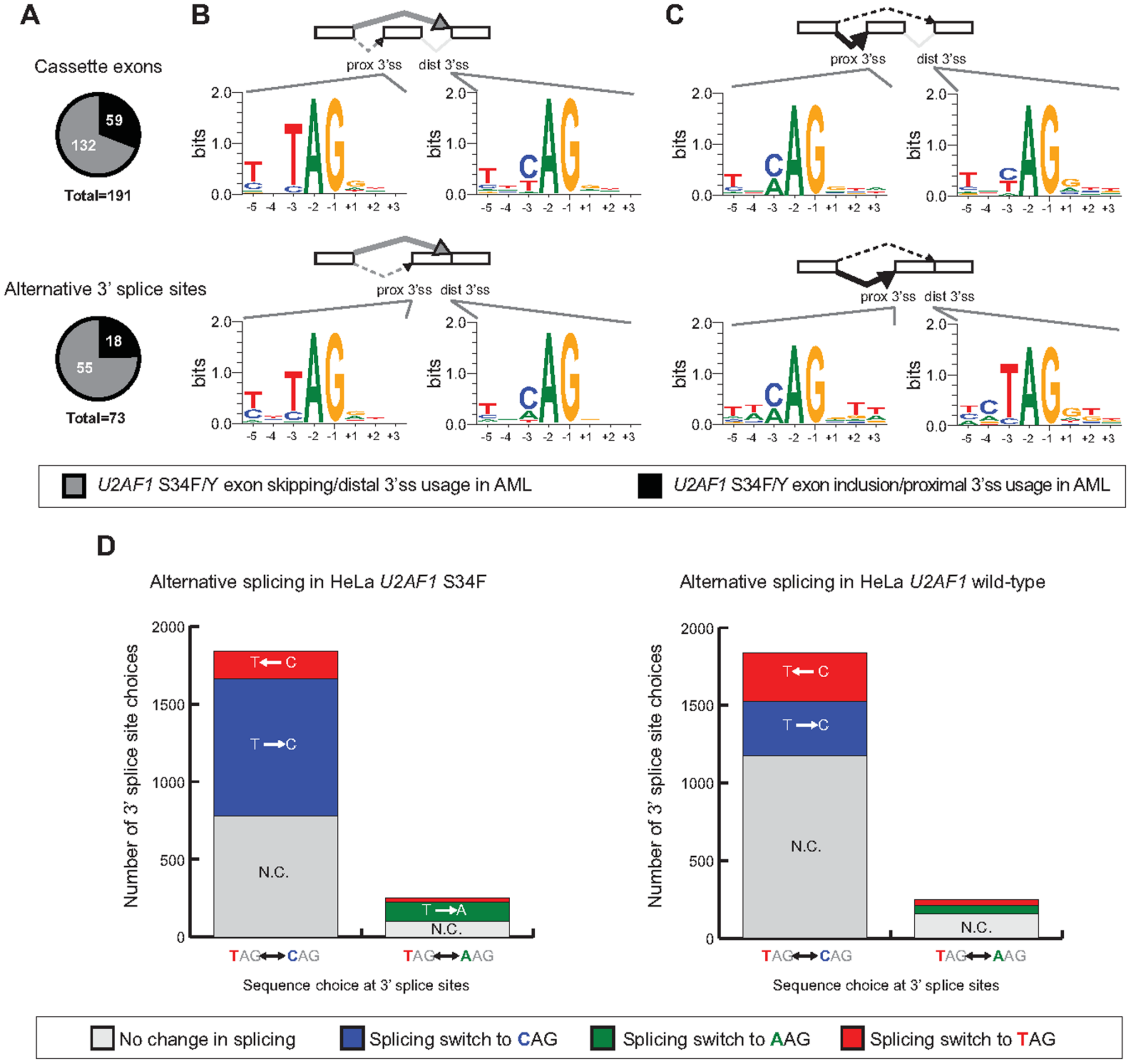
Sequence preferences at altered 3′ splice sites associated with *U2AF1* S34F/Y mutation. (A) Proportion of altered cassette exon and alternative 3′ splice site events that show exon skipping versus exon inclusion. (B) Consensus sequence motifs identified at the proximal (prox 3′ss) and distal 3′ splice sites (dist 3′ss) of exon skipping events. (C) Consensus sequence motifs at the proximal and distal 3′ splice sites of exon inclusion events. Splicing changes of expressed and annotated 3′ splice site choices where the splice site choice is TAG vs. CAG or TAG vs. AAG in (D) HeLa cells+induced *U2AF1* S34F or (E) HeLa cells+induced *U2AF1* wild-type.
