# Supplementary material for: A Pan-Cancer Analysis of Transcriptome Changes Associated with Somatic Mutations in U2AF1 Reveals Commonly Altered Splicing Events
Source: PLoS One. 2014 Jan 31;9(1):e87361. doi: 10.1371/journal.pone.0087361 (PMC3909098; doi:10.1371/journal.pone.0087361)
Supplement: Document S1 — Supporting figures and tables. Figure S1, Correlation of ΔPSI values between JuncBASE and Przychodzen et al. [1]. Figure S2, Highly variable alternative splicing events in lung adenocarcinoma. Figure S3, Splice site motifs at cassette exon and alternative 3′ splice site changes associated with U2AF1 S34F mutation in lung adenocarcinoma. Figure S4, Splice site motifs at cassette exon and alternative 3′ splice site changes associated with RBM10 loss-of-function (LOF) mutation in lung adenocarcinoma and control alternative splicing events. Figure S5, Splice site motifs at cassette exon and alternative 3′ splice site changes associated with induction of U2AF1 S34F or U2AF1 wild-type in HeLa cells. Figure S6, GSEA enrichment analysis in AML samples. Figure S7, Expression of V5 tagged U2AF1 WT or U2AF1 S34F constructs in 293T cells as determined by western blot. Table S1, The Cancer Genome Atlas sample identifiers used in this study. Somatic mutations in splicing factors are indicated. Table S2, Splicing factors reported to be significantly altered in previous studies where U2AF1 is somatically mutated. Table S3, Mitotic cell cycle genes that show differential splicing in the presence of the U2AF1 S34F/Y mutation. Table S4, Cancer Gene Census genes differentially spliced in the presence of a U2AF1 S34F/Y mutation. Table S5, Taqman assays (Life Technologies) used for quantitative RT-PCR of splicing events in CHCHD7, CHEK2, CTNNB1, KARS, and, PTBP1. (PDF) [file pone.0087361.s001.pdf]

Supporting Information

A pan-cancer analysis of transcriptome alterations associated with somatic mutations in *U2AF1* reveals commonly altered splicing events

Angela N. Brooks[1,2], Peter S. Choi[1,2], Luc de Waal[1,2], Tanaz Sharifnia[1,2], Marcin Imielinski[1,2], Gordon Saksena[1], Chandra Sekhar Pedamallu[1,2], Andrey Sivachenko[1], Mara Rosenberg[1], Juliann Chmielecki[1,2], Michael S. Lawrence[1], David S. DeLuca[1], Gad Getz[1], Matthew Meyerson[1,2,3]

[1]Cancer Program, Broad Institute of Harvard and Massachusetts Institute of Technology, Cambridge, Massachusetts, United States of America
[2]Department of Medical Oncology, Dana-Farber Cancer Institute, Boston, Massachusetts, United States of America
[3]Department of Pathology, Harvard Medical School, Boston, Massachusetts, United States of America

Table of Contents

SUPPORTING FIGURES **3**

Figure S1: Correlation of ΔPSI values between JuncBASE and Przychodzen *et al.* [1] **3**

Figure S2: Highly variable alternative splicing events in lung adenocarcinoma. **4**

Figure S3: Splice site motifs at cassette exon and alternative 3' splice site changes associated with *U2AF1* S34F mutation in lung adenocarcinoma. **5**

Figure S4: Splice site motifs at cassette exon and alternative 3' splice site changes associated with *RBM10* loss-of-function (LOF) mutation in lung adenocarcinoma and control alternative splicing events. **6**

Figure S5: Splice site motifs at cassette exon and alternative 3' splice site changes associated with induction of *U2AF1* S34F or *U2AF1* wild-type in HeLa cells. **7**

Figure S6: GSEA enrichment analysis in AML samples. **8**

Figure S7: Expression of V5 tagged U2AF1 WT or U2AF1 S34F constructs in 293T cells as determined by western blot **9**

SUPPORTING TABLES **10**

Table S1: The Cancer Genome Atlas sample identifiers used in this study. Somatic mutations in splicing factors are indicated. **10**

Table S2: Splicing factors reported to be significantly altered in previous studies where *U2AF1* is somatically mutated. **21**

Table S3: Mitotic cell cycle genes that show differential splicing in the presence of the *U2AF1* S34F/Y mutation. **22**

Table S4: Cancer Gene Census genes differentially spliced in the presence of a *U2AF1* S34F/Y mutation. **23**

Table S5: Taqman assays (Life Technologies) used for quantitative RT-PCR of splicing events in *CHCHD7, CHEK2, CTNNB1, KARS,* and, *PTBP1*. **24**

REFERENCES **25**

Supporting Figures

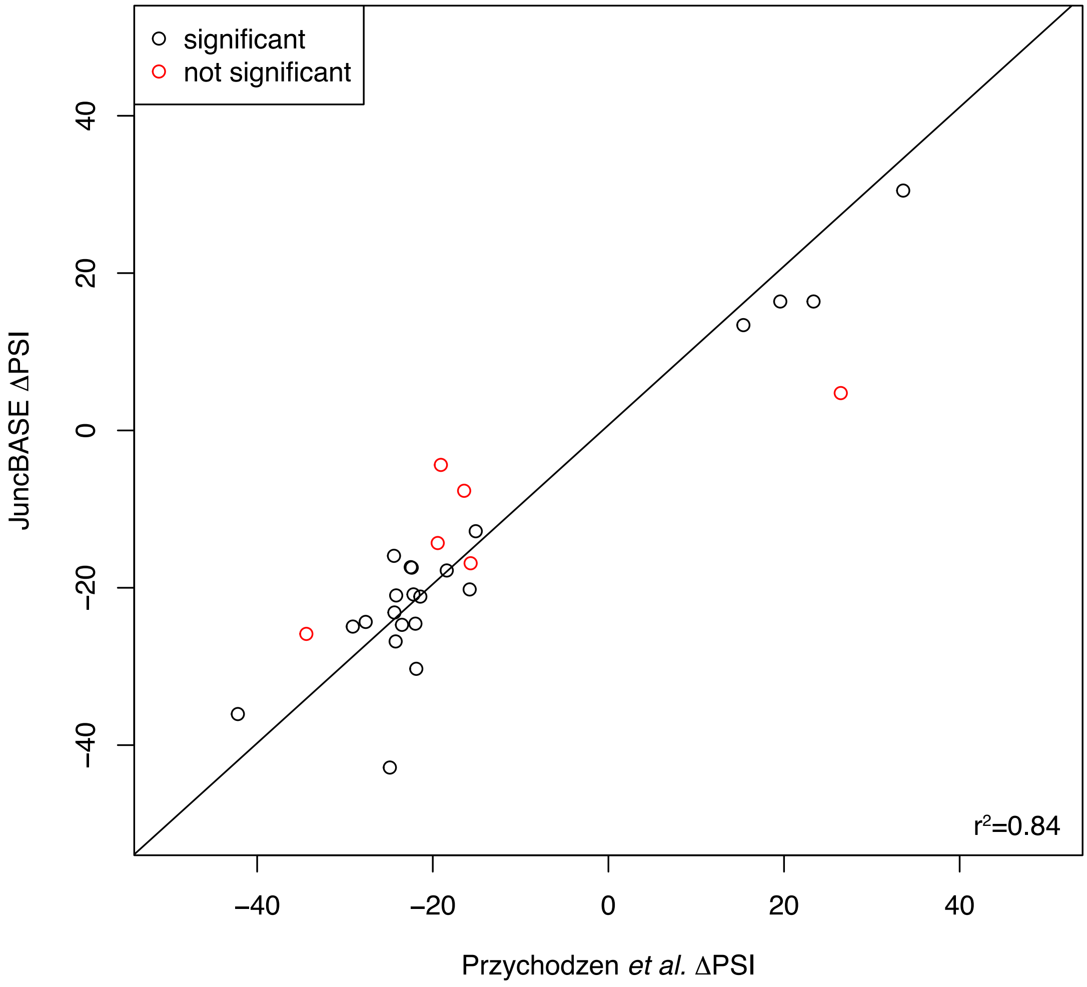

Figure S1: Correlation of ΔPSI values between JuncBASE and Przychodzen *et al.* [1]

Difference of percent spliced in (PSI) values between AML samples with and without splicing factor mutations were reported in Przychodzen *et al.* and compared to JuncBASE quantified values. Red circles indicate splice events that were quantified, but not significantly associated with *U2AF1* mutation by JuncBASE analysis. Least squares regression line is shown.

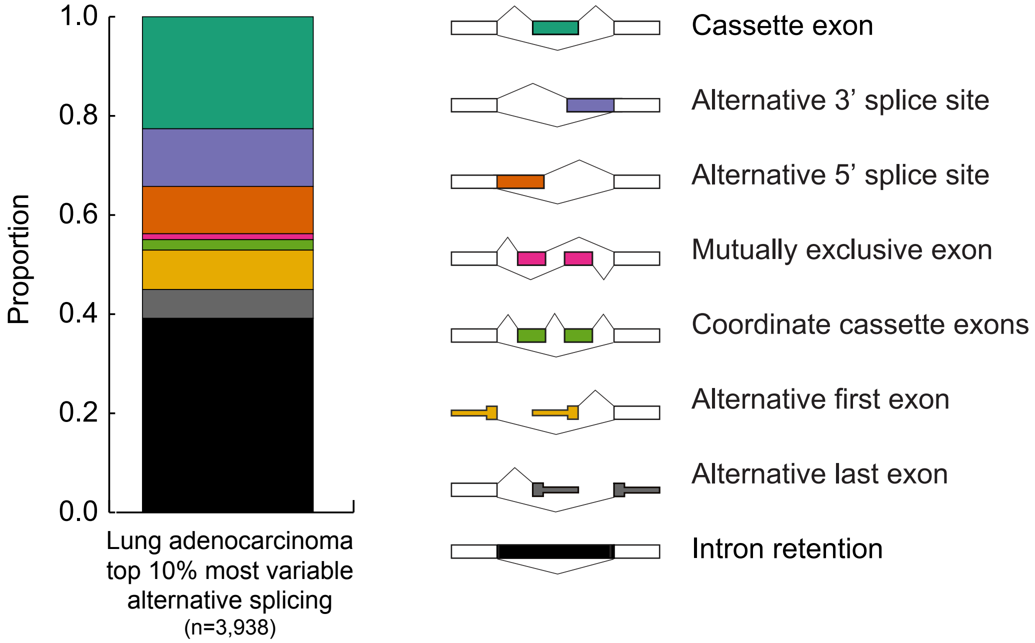

Figure S2: Highly variable alternative splicing events in lung adenocarcinoma.

Proportion of alternative splicing events in the top 10% most variable splicing events in lung adenocarcinomas with no mutation in a splicing factor.

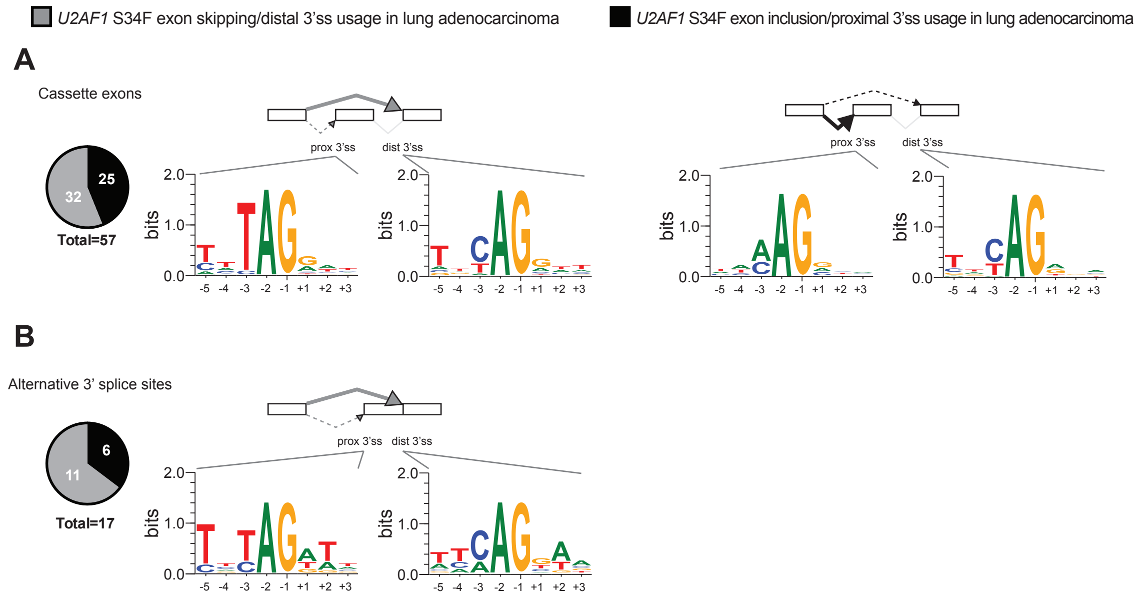

Figure S3: Splice site motifs at cassette exon and alternative 3' splice site changes associated with *U2AF1* S34F mutation in lung adenocarcinoma.

(**A**) Number of cassette exons showing significant skipping or inclusion in *U2AF1* mutated lung adenocarcinomas. Consensus motifs associated with the splice sites are shown. (**B**) Number of alternative 3' splice site events showing significant skipping or inclusion in *U2AF1* mutated lung adenocarcinomas. Consensus motifs associated with the splice sites are shown. Consensus motifs were only made for cases with 10 or more exon skipping or inclusion events.

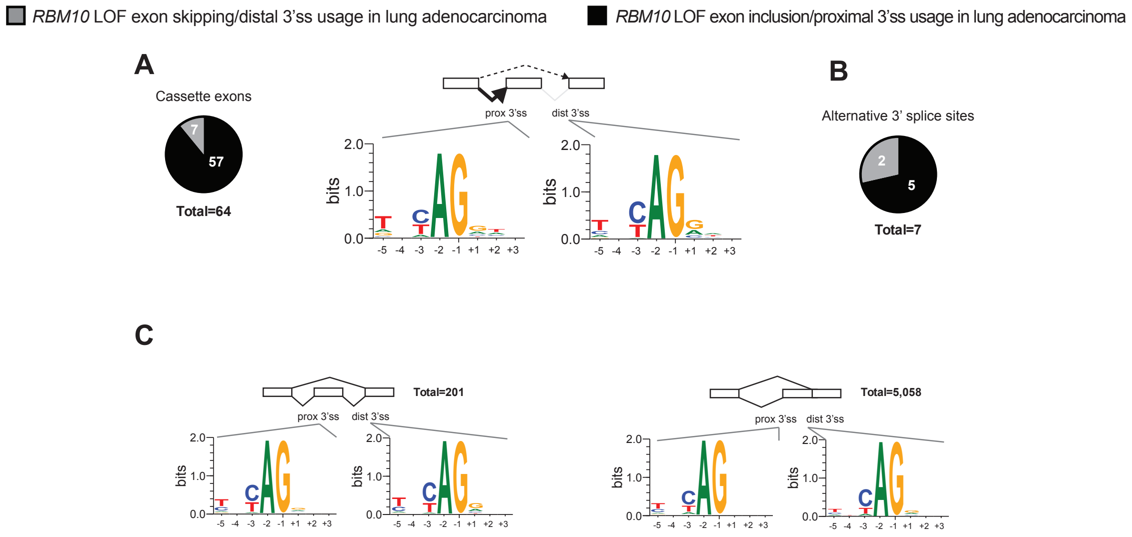

Figure S4: Splice site motifs at cassette exon and alternative 3' splice site changes associated with *RBM10* loss-of-function (LOF) mutation in lung adenocarcinoma and control alternative splicing events.

(**A**) Number of cassette exons showing significant skipping or inclusion in *RBM10* LOF lung adenocarcinomas. Consensus motifs associated with the splice sites are shown. (**B**) Number of alternative 3' splice site events showing significant skipping or inclusion in *RBM10* LOF lung adenocarcinomas. Consensus motifs were only made for cases with 10 or more exon skipping or inclusion events. (**C**) Consensus sequence motifs at the proximal and distal 3' splice sites of control alternative splicing events.

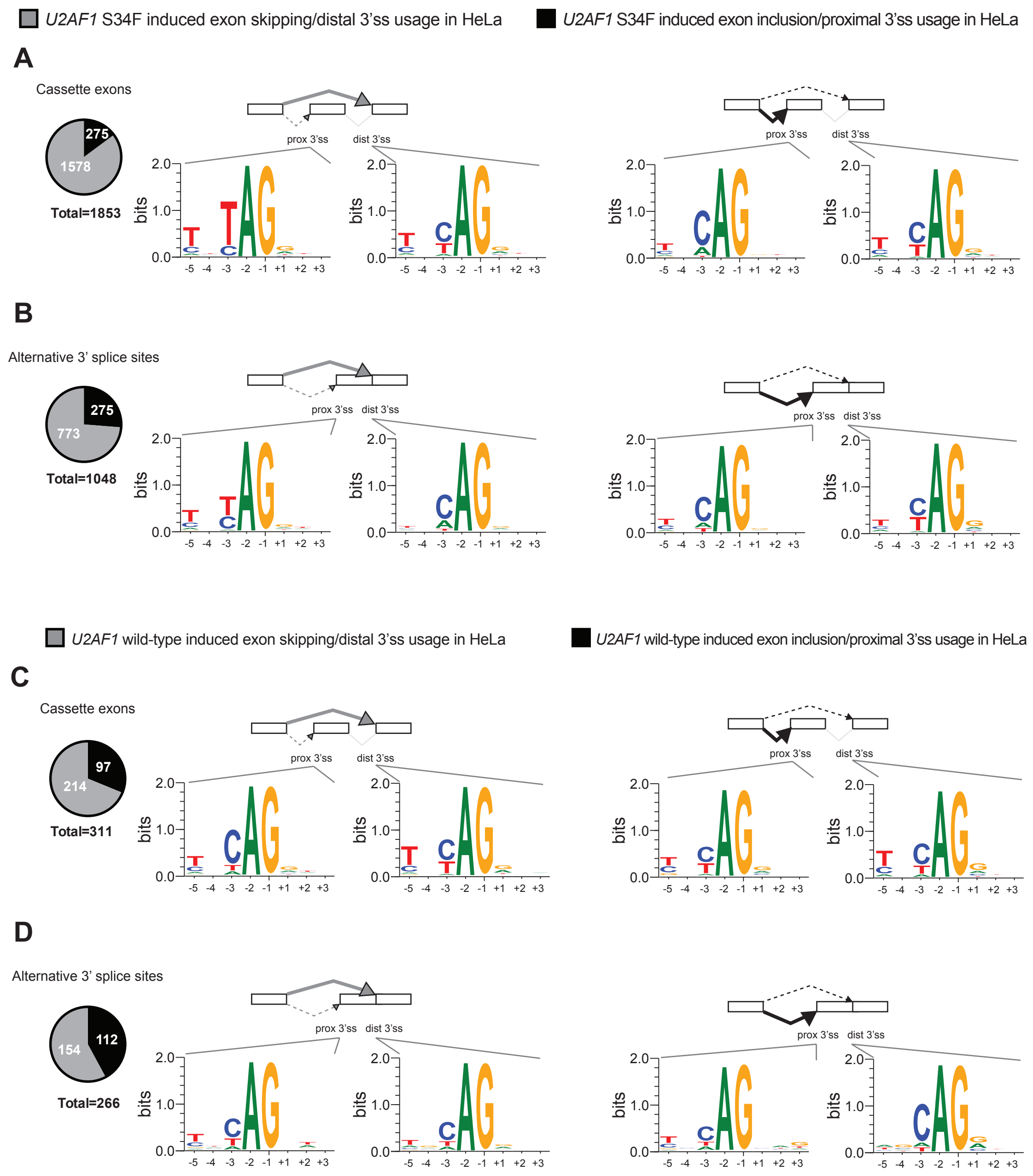

Figure S5: Splice site motifs at cassette exon and alternative 3' splice site changes associated with induction of *U2AF1* S34F or *U2AF1* wild-type in HeLa cells.

Number of cassette exons showing significant skipping or inclusion in HeLa cells + induction of (**A**) *U2AF1* S34F or (**C**) U2AF1 wild-type. Number of alternative 3' splice sites showing significant skipping or inclusion in HeLa cells + induction of (**B**) *U2AF1* S34F or (**D**) U2AF1 wild-type. Consensus motifs associated with the splice sites are shown.

A

GO:0000278
mitotic cell cycle

Enrichment score:
0.582

Normalized enrichment score:
1.735

Nominal p-value:
0.038

FDR:
1

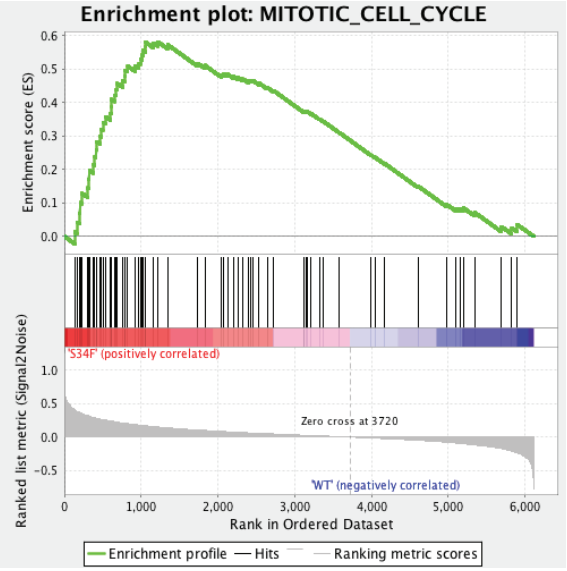

B

GO:0000087
M phase of mitotic cell cycle

Enrichment score:
0.688

Normalized enrichment score:
1.679

Nominal p-value:
0.048

FDR:
0.968

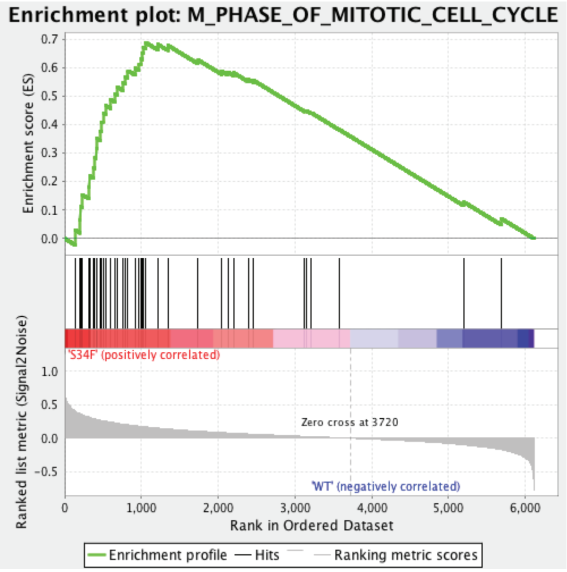

Figure S6: GSEA enrichment analysis in AML samples.

(**A**) GO:0000278 mitotic cell cycle enrichment score. (**B**) GO:0000087 M phase of mitotic cell cycle enrichment score.

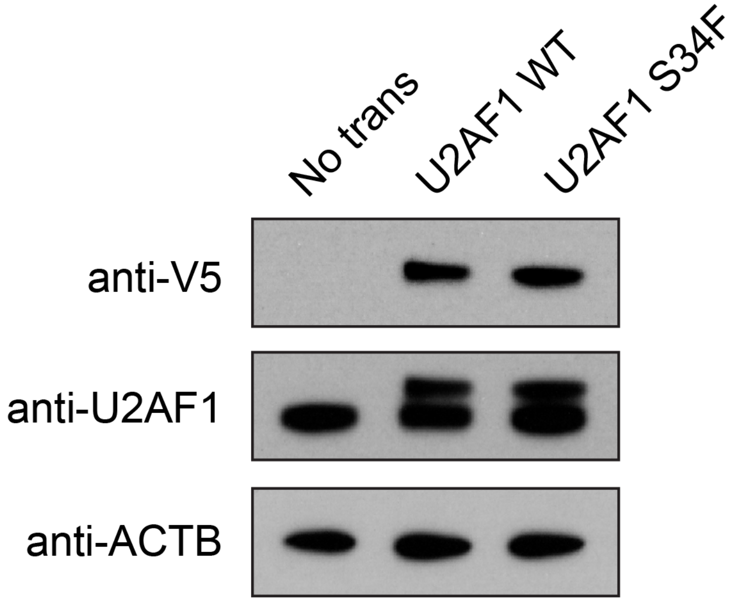

Figure S7: Expression of V5 tagged U2AF1 WT or U2AF1 S34F constructs in 293T cells as determined by western blot

Supporting Tables

Table S1: The Cancer Genome Atlas sample identifiers used in this study. Somatic mutations in splicing factors are indicated.

LUAD, lung adenocarcinoma; LAML, acute myeloid leukemia

TCGA ID	cancer type	U2AF1	RBM10	ZRSR2	U2AF2	SF3B1	PRPF40B	SF1	SF3A1	HNRNPK
TCGA-55-7727	LUAD	p.S34F	p.Q416L	WT	WT	WT	WT	WT	WT	WT
TCGA-64-1680	LUAD	p.S34F	WT	WT	WT	WT	WT	p.Y450*	WT	WT
TCGA-49-6744	LUAD	p.S34F	WT	WT	WT	WT	WT	WT	WT	WT
TCGA-55-7903	LUAD	p.S34F	WT	WT	WT	WT	WT	WT	WT	WT
TCGA-50-5941	LUAD	p.S34F	WT	WT	WT	WT	WT	WT	WT	WT
TCGA-78-7145	LUAD	p.S34F	WT	WT	WT	WT	WT	WT	WT	WT
TCGA-49-4505	LUAD	p.S34F	WT	WT	WT	WT	WT	WT	WT	WT
TCGA-49-4488	LUAD	p.S34F	WT	WT	WT	WT	WT	WT	WT	WT
TCGA-55-7815	LUAD	WT	p.D568fs	WT	WT	WT	WT	WT	WT	WT
TCGA-05-4424	LUAD	WT	p.E177*	WT	WT	WT	WT	WT	WT	WT
TCGA-05-4418	LUAD	WT	p.E559*	WT	WT	WT	WT	WT	WT	WT
TCGA-78-7158	LUAD	WT	p.E800*	WT	WT	WT	WT	WT	WT	WT
TCGA-64-1676	LUAD	WT	p.G153C	WT	WT	p.A959V	WT	WT	WT	WT
TCGA-78-7148	LUAD	WT	p.G168_splice	WT	WT	WT	WT	WT	WT	WT
TCGA-75-6214	LUAD	WT	p.G168_splice	WT	WT	WT	WT	WT	WT	WT
TCGA-55-7281	LUAD	WT	p.G870C	WT	WT	WT	WT	WT	WT	WT
TCGA-64-5775	LUAD	WT	p.P259fs	WT	WT	WT	WT	WT	WT	WT
TCGA-55-6983	LUAD	WT	p.P567fs	WT	WT	WT	WT	WT	WT	WT
TCGA-05-4403	LUAD	WT	p.Q155*	WT	WT	WT	WT	WT	WT	WT
TCGA-49-4494	LUAD	WT	p.Q192_splice	WT	WT	WT	WT	WT	WT	WT
TCGA-44-7659	LUAD	WT	p.Q674*	WT	WT	WT	WT	WT	WT	WT
TCGA-78-7537	LUAD	WT	p.R163fs	WT	WT	WT	WT	WT	WT	WT
TCGA-73-7498	LUAD	WT	p.R387_splice	WT	WT	WT	WT	WT	WT	WT
TCGA-69-7764	LUAD	WT	p.S781S	WT	WT	WT	WT	WT	WT	WT
TCGA-35-3615	LUAD	WT	p.T185fs	WT	WT	WT	WT	WT	WT	WT
TCGA-05-4395	LUAD	WT	p.W658Y	WT	WT	WT	WT	WT	WT	WT
TCGA-78-7166	LUAD	WT	p.Y16fs	WT	WT	WT	WT	WT	WT	WT
TCGA-97-7938	LUAD	WT	WT	p.A342S	WT	WT	WT	WT	WT	WT
TCGA-75-6211	LUAD	WT	WT	WT	p.A131S	WT	WT	WT	WT	WT
TCGA-55-6543	LUAD	WT	WT	WT	p.F4Y	WT	WT	WT	WT	WT
TCGA-38-4627	LUAD	WT	WT	WT	p.G176E	WT	WT	WT	WT	WT

TCGA-50-5933	LUAD	WT	WT	WT	p.G176V	WT	WT	WT	WT	WT
TCGA-69-7980	LUAD	WT	WT	WT	p.I263I	WT	WT	WT	WT	WT
TCGA-97-7554	LUAD	WT	WT	WT	p.P361P	WT	p.R410L	WT	WT	WT
TCGA-67-3772	LUAD	WT	WT	WT	p.P95fs	WT	WT	WT	WT	WT
TCGA-55-7574	LUAD	WT	WT	WT	p.V291V	WT	WT	WT	WT	WT
TCGA-44-6774	LUAD	WT	WT	WT	WT	p.A899S	WT	WT	WT	WT
TCGA-99-7458	LUAD	WT	WT	WT	WT	p.G237G	WT	WT	WT	WT
TCGA-44-2659	LUAD	WT	WT	WT	WT	p.K741N	WT	WT	WT	WT
TCGA-50-5936	LUAD	WT	WT	WT	WT	p.P279S	WT	WT	WT	WT
TCGA-49-4487	LUAD	WT	WT	WT	WT	p.R292K	WT	WT	WT	WT
TCGA-91-7771	LUAD	WT	WT	WT	WT	WT	p.R365S	WT	WT	WT
TCGA-64-5778	LUAD	WT	WT	WT	WT	WT	p.S766F	WT	WT	WT
TCGA-50-6597	LUAD	WT	WT	WT	WT	WT	WT	p.A551A	WT	WT
TCGA-38-4631	LUAD	WT	WT	WT	WT	WT	WT	p.N120S	WT	WT
TCGA-05-4422	LUAD	WT	WT	WT	WT	WT	WT	p.P444S	WT	WT
TCGA-38-4632	LUAD	WT	WT	WT	WT	WT	WT	p.S268L	WT	WT
TCGA-78-7539	LUAD	WT	WT	WT	WT	WT	WT	WT	p.D196G	WT
TCGA-97-7546	LUAD	WT	WT	WT	WT	WT	WT	WT	p.E340*	WT
TCGA-05-4417	LUAD	WT	WT	WT	WT	WT	WT	WT	p.G489C	WT
TCGA-86-6851	LUAD	WT	WT	WT	WT	WT	WT	WT	p.P414H	WT
TCGA-44-7670	LUAD	WT	WT	WT	WT	WT	WT	WT	p.S227*	WT
TCGA-05-4390	LUAD	WT	WT	WT	WT	WT	WT	WT	p.Y271C	WT
TCGA-55-1592	LUAD	WT	WT	WT	WT	WT	WT	WT	WT	p.Q31R
TCGA-55-6982	LUAD	WT	WT	WT	WT	WT	WT	WT	WT	p.R221H
TCGA-05-4249	LUAD	WT	WT	WT	WT	WT	WT	WT	WT	c.-38A>G
TCGA-AB-2821	LAML	p.Q157P	WT	WT	WT	WT	WT	WT	WT	WT
TCGA-AB-2996	LAML	p.S34F	WT	WT	WT	WT	WT	WT	WT	WT
TCGA-AB-2847	LAML	p.S34F	WT	WT	WT	WT	WT	WT	WT	WT
TCGA-AB-2843	LAML	p.S34F	WT	WT	WT	WT	WT	WT	WT	WT
TCGA-AB-2912	LAML	p.S34F	WT	WT	WT	WT	WT	WT	WT	WT
TCGA-AB-2882	LAML	p.S34Y	WT	WT	WT	WT	WT	WT	WT	WT
TCGA-AB-2861	LAML	p.S34Y	WT	WT	WT	WT	WT	WT	WT	WT
TCGA-AB-2851	LAML	WT	WT	WT	p.L14fs	WT	WT	WT	WT	WT
TCGA-AB-2929	LAML	WT	WT	WT	WT	p.K700E	WT	WT	WT	WT
TCGA-AB-2859	LAML	WT	WT	WT	WT	WT	WT	WT	WT	p.L68fs
TCGA-AB-2937	LAML	WT	WT	WT	WT	WT	WT	WT	WT	p.N72fs
TCGA-05-4382	LUAD	WT	WT	WT	WT	WT	WT	WT	WT	WT
TCGA-05-4384	LUAD	WT	WT	WT	WT	WT	WT	WT	WT	WT
TCGA-05-4389	LUAD	WT	WT	WT	WT	WT	WT	WT	WT	WT

TCGA-05-4396	LUAD	WT	WT	WT	WT	WT	WT	WT	WT	WT
TCGA-05-4398	LUAD	WT	WT	WT	WT	WT	WT	WT	WT	WT
TCGA-05-4402	LUAD	WT	WT	WT	WT	WT	WT	WT	WT	WT
TCGA-05-4405	LUAD	WT	WT	WT	WT	WT	WT	WT	WT	WT
TCGA-05-4410	LUAD	WT	WT	WT	WT	WT	WT	WT	WT	WT
TCGA-05-4415	LUAD	WT	WT	WT	WT	WT	WT	WT	WT	WT
TCGA-05-4420	LUAD	WT	WT	WT	WT	WT	WT	WT	WT	WT
TCGA-05-4425	LUAD	WT	WT	WT	WT	WT	WT	WT	WT	WT
TCGA-05-4426	LUAD	WT	WT	WT	WT	WT	WT	WT	WT	WT
TCGA-05-4430	LUAD	WT	WT	WT	WT	WT	WT	WT	WT	WT
TCGA-05-4432	LUAD	WT	WT	WT	WT	WT	WT	WT	WT	WT
TCGA-05-4433	LUAD	WT	WT	WT	WT	WT	WT	WT	WT	WT
TCGA-05-5420	LUAD	WT	WT	WT	WT	WT	WT	WT	WT	WT
TCGA-05-5423	LUAD	WT	WT	WT	WT	WT	WT	WT	WT	WT
TCGA-05-5428	LUAD	WT	WT	WT	WT	WT	WT	WT	WT	WT
TCGA-05-5429	LUAD	WT	WT	WT	WT	WT	WT	WT	WT	WT
TCGA-05-5715	LUAD	WT	WT	WT	WT	WT	WT	WT	WT	WT
TCGA-38-4626	LUAD	WT	WT	WT	WT	WT	WT	WT	WT	WT
TCGA-38-4628	LUAD	WT	WT	WT	WT	WT	WT	WT	WT	WT
TCGA-38-6178	LUAD	WT	WT	WT	WT	WT	WT	WT	WT	WT
TCGA-44-2655	LUAD	WT	WT	WT	WT	WT	WT	WT	WT	WT
TCGA-44-2656	LUAD	WT	WT	WT	WT	WT	WT	WT	WT	WT
TCGA-44-2657	LUAD	WT	WT	WT	WT	WT	WT	WT	WT	WT
TCGA-44-2661	LUAD	WT	WT	WT	WT	WT	WT	WT	WT	WT
TCGA-44-2662	LUAD	WT	WT	WT	WT	WT	WT	WT	WT	WT
TCGA-44-2665	LUAD	WT	WT	WT	WT	WT	WT	WT	WT	WT
TCGA-44-2666	LUAD	WT	WT	WT	WT	WT	WT	WT	WT	WT
TCGA-44-3396	LUAD	WT	WT	WT	WT	WT	WT	WT	WT	WT
TCGA-44-3919	LUAD	WT	WT	WT	WT	WT	WT	WT	WT	WT
TCGA-44-4112	LUAD	WT	WT	WT	WT	WT	WT	WT	WT	WT
TCGA-44-5645	LUAD	WT	WT	WT	WT	WT	WT	WT	WT	WT
TCGA-44-6145	LUAD	WT	WT	WT	WT	WT	WT	WT	WT	WT
TCGA-44-6146	LUAD	WT	WT	WT	WT	WT	WT	WT	WT	WT
TCGA-44-6147	LUAD	WT	WT	WT	WT	WT	WT	WT	WT	WT
TCGA-44-6775	LUAD	WT	WT	WT	WT	WT	WT	WT	WT	WT
TCGA-44-6776	LUAD	WT	WT	WT	WT	WT	WT	WT	WT	WT
TCGA-44-6777	LUAD	WT	WT	WT	WT	WT	WT	WT	WT	WT
TCGA-44-6779	LUAD	WT	WT	WT	WT	WT	WT	WT	WT	WT
TCGA-44-7662	LUAD	WT	WT	WT	WT	WT	WT	WT	WT	WT

TCGA-44-7671	LUAD	WT	WT	WT	WT	WT	WT	WT	WT	WT
TCGA-44-7672	LUAD	WT	WT	WT	WT	WT	WT	WT	WT	WT
TCGA-49-4486	LUAD	WT	WT	WT	WT	WT	WT	WT	WT	WT
TCGA-49-4490	LUAD	WT	WT	WT	WT	WT	WT	WT	WT	WT
TCGA-49-4501	LUAD	WT	WT	WT	WT	WT	WT	WT	WT	WT
TCGA-49-4506	LUAD	WT	WT	WT	WT	WT	WT	WT	WT	WT
TCGA-49-4507	LUAD	WT	WT	WT	WT	WT	WT	WT	WT	WT
TCGA-49-4510	LUAD	WT	WT	WT	WT	WT	WT	WT	WT	WT
TCGA-49-4512	LUAD	WT	WT	WT	WT	WT	WT	WT	WT	WT
TCGA-49-4514	LUAD	WT	WT	WT	WT	WT	WT	WT	WT	WT
TCGA-49-6742	LUAD	WT	WT	WT	WT	WT	WT	WT	WT	WT
TCGA-49-6745	LUAD	WT	WT	WT	WT	WT	WT	WT	WT	WT
TCGA-49-6761	LUAD	WT	WT	WT	WT	WT	WT	WT	WT	WT
TCGA-49-6767	LUAD	WT	WT	WT	WT	WT	WT	WT	WT	WT
TCGA-50-5044	LUAD	WT	WT	WT	WT	WT	WT	WT	WT	WT
TCGA-50-5049	LUAD	WT	WT	WT	WT	WT	WT	WT	WT	WT
TCGA-50-5051	LUAD	WT	WT	WT	WT	WT	WT	WT	WT	WT
TCGA-50-5055	LUAD	WT	WT	WT	WT	WT	WT	WT	WT	WT
TCGA-50-5068	LUAD	WT	WT	WT	WT	WT	WT	WT	WT	WT
TCGA-50-5072	LUAD	WT	WT	WT	WT	WT	WT	WT	WT	WT
TCGA-50-5931	LUAD	WT	WT	WT	WT	WT	WT	WT	WT	WT
TCGA-50-5932	LUAD	WT	WT	WT	WT	WT	WT	WT	WT	WT
TCGA-50-5935	LUAD	WT	WT	WT	WT	WT	WT	WT	WT	WT
TCGA-50-5939	LUAD	WT	WT	WT	WT	WT	WT	WT	WT	WT
TCGA-50-5942	LUAD	WT	WT	WT	WT	WT	WT	WT	WT	WT
TCGA-50-5944	LUAD	WT	WT	WT	WT	WT	WT	WT	WT	WT
TCGA-50-6593	LUAD	WT	WT	WT	WT	WT	WT	WT	WT	WT
TCGA-50-6595	LUAD	WT	WT	WT	WT	WT	WT	WT	WT	WT
TCGA-50-6673	LUAD	WT	WT	WT	WT	WT	WT	WT	WT	WT
TCGA-50-7109	LUAD	WT	WT	WT	WT	WT	WT	WT	WT	WT
TCGA-53-7626	LUAD	WT	WT	WT	WT	WT	WT	WT	WT	WT
TCGA-53-7813	LUAD	WT	WT	WT	WT	WT	WT	WT	WT	WT
TCGA-55-1594	LUAD	WT	WT	WT	WT	WT	WT	WT	WT	WT
TCGA-55-1596	LUAD	WT	WT	WT	WT	WT	WT	WT	WT	WT
TCGA-55-6642	LUAD	WT	WT	WT	WT	WT	WT	WT	WT	WT
TCGA-55-6712	LUAD	WT	WT	WT	WT	WT	WT	WT	WT	WT
TCGA-55-6970	LUAD	WT	WT	WT	WT	WT	WT	WT	WT	WT
TCGA-55-6971	LUAD	WT	WT	WT	WT	WT	WT	WT	WT	WT
TCGA-55-6972	LUAD	WT	WT	WT	WT	WT	WT	WT	WT	WT

TCGA-55-6978	LUAD	WT	WT	WT	WT	WT	WT	WT	WT	WT
TCGA-55-6979	LUAD	WT	WT	WT	WT	WT	WT	WT	WT	WT
TCGA-55-6980	LUAD	WT	WT	WT	WT	WT	WT	WT	WT	WT
TCGA-55-6981	LUAD	WT	WT	WT	WT	WT	WT	WT	WT	WT
TCGA-55-6985	LUAD	WT	WT	WT	WT	WT	WT	WT	WT	WT
TCGA-55-6986	LUAD	WT	WT	WT	WT	WT	WT	WT	WT	WT
TCGA-55-7283	LUAD	WT	WT	WT	WT	WT	WT	WT	WT	WT
TCGA-55-7573	LUAD	WT	WT	WT	WT	WT	WT	WT	WT	WT
TCGA-55-7576	LUAD	WT	WT	WT	WT	WT	WT	WT	WT	WT
TCGA-55-7724	LUAD	WT	WT	WT	WT	WT	WT	WT	WT	WT
TCGA-55-7725	LUAD	WT	WT	WT	WT	WT	WT	WT	WT	WT
TCGA-55-7726	LUAD	WT	WT	WT	WT	WT	WT	WT	WT	WT
TCGA-55-7728	LUAD	WT	WT	WT	WT	WT	WT	WT	WT	WT
TCGA-55-7907	LUAD	WT	WT	WT	WT	WT	WT	WT	WT	WT
TCGA-55-7911	LUAD	WT	WT	WT	WT	WT	WT	WT	WT	WT
TCGA-55-7914	LUAD	WT	WT	WT	WT	WT	WT	WT	WT	WT
TCGA-64-1677	LUAD	WT	WT	WT	WT	WT	WT	WT	WT	WT
TCGA-64-1678	LUAD	WT	WT	WT	WT	WT	WT	WT	WT	WT
TCGA-64-1679	LUAD	WT	WT	WT	WT	WT	WT	WT	WT	WT
TCGA-64-1681	LUAD	WT	WT	WT	WT	WT	WT	WT	WT	WT
TCGA-64-5774	LUAD	WT	WT	WT	WT	WT	WT	WT	WT	WT
TCGA-64-5781	LUAD	WT	WT	WT	WT	WT	WT	WT	WT	WT
TCGA-67-3771	LUAD	WT	WT	WT	WT	WT	WT	WT	WT	WT
TCGA-67-3773	LUAD	WT	WT	WT	WT	WT	WT	WT	WT	WT
TCGA-67-3774	LUAD	WT	WT	WT	WT	WT	WT	WT	WT	WT
TCGA-67-6215	LUAD	WT	WT	WT	WT	WT	WT	WT	WT	WT
TCGA-67-6216	LUAD	WT	WT	WT	WT	WT	WT	WT	WT	WT
TCGA-67-6217	LUAD	WT	WT	WT	WT	WT	WT	WT	WT	WT
TCGA-69-7760	LUAD	WT	WT	WT	WT	WT	WT	WT	WT	WT
TCGA-69-7761	LUAD	WT	WT	WT	WT	WT	WT	WT	WT	WT
TCGA-69-7763	LUAD	WT	WT	WT	WT	WT	WT	WT	WT	WT
TCGA-69-7765	LUAD	WT	WT	WT	WT	WT	WT	WT	WT	WT
TCGA-71-6725	LUAD	WT	WT	WT	WT	WT	WT	WT	WT	WT
TCGA-73-4658	LUAD	WT	WT	WT	WT	WT	WT	WT	WT	WT
TCGA-73-4659	LUAD	WT	WT	WT	WT	WT	WT	WT	WT	WT
TCGA-73-4662	LUAD	WT	WT	WT	WT	WT	WT	WT	WT	WT
TCGA-73-4675	LUAD	WT	WT	WT	WT	WT	WT	WT	WT	WT
TCGA-73-4676	LUAD	WT	WT	WT	WT	WT	WT	WT	WT	WT
TCGA-73-7499	LUAD	WT	WT	WT	WT	WT	WT	WT	WT	WT

TCGA-75-5122	LUAD	WT	WT	WT	WT	WT	WT	WT	WT	WT
TCGA-75-5126	LUAD	WT	WT	WT	WT	WT	WT	WT	WT	WT
TCGA-75-5146	LUAD	WT	WT	WT	WT	WT	WT	WT	WT	WT
TCGA-75-5147	LUAD	WT	WT	WT	WT	WT	WT	WT	WT	WT
TCGA-75-6203	LUAD	WT	WT	WT	WT	WT	WT	WT	WT	WT
TCGA-75-6205	LUAD	WT	WT	WT	WT	WT	WT	WT	WT	WT
TCGA-75-6207	LUAD	WT	WT	WT	WT	WT	WT	WT	WT	WT
TCGA-75-6212	LUAD	WT	WT	WT	WT	WT	WT	WT	WT	WT
TCGA-75-7025	LUAD	WT	WT	WT	WT	WT	WT	WT	WT	WT
TCGA-75-7027	LUAD	WT	WT	WT	WT	WT	WT	WT	WT	WT
TCGA-75-7030	LUAD	WT	WT	WT	WT	WT	WT	WT	WT	WT
TCGA-75-7031	LUAD	WT	WT	WT	WT	WT	WT	WT	WT	WT
TCGA-78-7143	LUAD	WT	WT	WT	WT	WT	WT	WT	WT	WT
TCGA-78-7147	LUAD	WT	WT	WT	WT	WT	WT	WT	WT	WT
TCGA-78-7149	LUAD	WT	WT	WT	WT	WT	WT	WT	WT	WT
TCGA-78-7150	LUAD	WT	WT	WT	WT	WT	WT	WT	WT	WT
TCGA-78-7152	LUAD	WT	WT	WT	WT	WT	WT	WT	WT	WT
TCGA-78-7153	LUAD	WT	WT	WT	WT	WT	WT	WT	WT	WT
TCGA-78-7154	LUAD	WT	WT	WT	WT	WT	WT	WT	WT	WT
TCGA-78-7155	LUAD	WT	WT	WT	WT	WT	WT	WT	WT	WT
TCGA-78-7156	LUAD	WT	WT	WT	WT	WT	WT	WT	WT	WT
TCGA-78-7159	LUAD	WT	WT	WT	WT	WT	WT	WT	WT	WT
TCGA-78-7160	LUAD	WT	WT	WT	WT	WT	WT	WT	WT	WT
TCGA-78-7161	LUAD	WT	WT	WT	WT	WT	WT	WT	WT	WT
TCGA-78-7162	LUAD	WT	WT	WT	WT	WT	WT	WT	WT	WT
TCGA-78-7163	LUAD	WT	WT	WT	WT	WT	WT	WT	WT	WT
TCGA-78-7167	LUAD	WT	WT	WT	WT	WT	WT	WT	WT	WT
TCGA-78-7535	LUAD	WT	WT	WT	WT	WT	WT	WT	WT	WT
TCGA-78-7536	LUAD	WT	WT	WT	WT	WT	WT	WT	WT	WT
TCGA-78-7540	LUAD	WT	WT	WT	WT	WT	WT	WT	WT	WT
TCGA-78-7542	LUAD	WT	WT	WT	WT	WT	WT	WT	WT	WT
TCGA-78-7633	LUAD	WT	WT	WT	WT	WT	WT	WT	WT	WT
TCGA-80-5607	LUAD	WT	WT	WT	WT	WT	WT	WT	WT	WT
TCGA-80-5608	LUAD	WT	WT	WT	WT	WT	WT	WT	WT	WT
TCGA-86-6562	LUAD	WT	WT	WT	WT	WT	WT	WT	WT	WT
TCGA-86-7713	LUAD	WT	WT	WT	WT	WT	WT	WT	WT	WT
TCGA-86-7714	LUAD	WT	WT	WT	WT	WT	WT	WT	WT	WT
TCGA-91-6828	LUAD	WT	WT	WT	WT	WT	WT	WT	WT	WT
TCGA-91-6829	LUAD	WT	WT	WT	WT	WT	WT	WT	WT	WT

TCGA-91-6835	LUAD	WT	WT	WT	WT	WT	WT	WT	WT	WT
TCGA-91-6840	LUAD	WT	WT	WT	WT	WT	WT	WT	WT	WT
TCGA-91-6847	LUAD	WT	WT	WT	WT	WT	WT	WT	WT	WT
TCGA-91-6849	LUAD	WT	WT	WT	WT	WT	WT	WT	WT	WT
TCGA-93-7347	LUAD	WT	WT	WT	WT	WT	WT	WT	WT	WT
TCGA-93-7348	LUAD	WT	WT	WT	WT	WT	WT	WT	WT	WT
TCGA-95-7039	LUAD	WT	WT	WT	WT	WT	WT	WT	WT	WT
TCGA-95-7043	LUAD	WT	WT	WT	WT	WT	WT	WT	WT	WT
TCGA-95-7567	LUAD	WT	WT	WT	WT	WT	WT	WT	WT	WT
TCGA-95-7947	LUAD	WT	WT	WT	WT	WT	WT	WT	WT	WT
TCGA-95-7948	LUAD	WT	WT	WT	WT	WT	WT	WT	WT	WT
TCGA-97-7552	LUAD	WT	WT	WT	WT	WT	WT	WT	WT	WT
TCGA-97-7553	LUAD	WT	WT	WT	WT	WT	WT	WT	WT	WT
TCGA-97-7937	LUAD	WT	WT	WT	WT	WT	WT	WT	WT	WT
TCGA-97-7941	LUAD	WT	WT	WT	WT	WT	WT	WT	WT	WT
TCGA-AB-2866	LAML	WT	WT	WT	WT	WT	WT	WT	WT	WT
TCGA-AB-2899	LAML	WT	WT	WT	WT	WT	WT	WT	WT	WT
TCGA-AB-2897	LAML	WT	WT	WT	WT	WT	WT	WT	WT	WT
TCGA-AB-2904	LAML	WT	WT	WT	WT	WT	WT	WT	WT	WT
TCGA-AB-2871	LAML	WT	WT	WT	WT	WT	WT	WT	WT	WT
TCGA-AB-2856	LAML	WT	WT	WT	WT	WT	WT	WT	WT	WT
TCGA-AB-2819	LAML	WT	WT	WT	WT	WT	WT	WT	WT	WT
TCGA-AB-2985	LAML	WT	WT	WT	WT	WT	WT	WT	WT	WT
TCGA-AB-2828	LAML	WT	WT	WT	WT	WT	WT	WT	WT	WT
TCGA-AB-2982	LAML	WT	WT	WT	WT	WT	WT	WT	WT	WT
TCGA-AB-2816	LAML	WT	WT	WT	WT	WT	WT	WT	WT	WT
TCGA-AB-2976	LAML	WT	WT	WT	WT	WT	WT	WT	WT	WT
TCGA-AB-2908	LAML	WT	WT	WT	WT	WT	WT	WT	WT	WT
TCGA-AB-2885	LAML	WT	WT	WT	WT	WT	WT	WT	WT	WT
TCGA-AB-2886	LAML	WT	WT	WT	WT	WT	WT	WT	WT	WT
TCGA-AB-2994	LAML	WT	WT	WT	WT	WT	WT	WT	WT	WT
TCGA-AB-2824	LAML	WT	WT	WT	WT	WT	WT	WT	WT	WT
TCGA-AB-2920	LAML	WT	WT	WT	WT	WT	WT	WT	WT	WT
TCGA-AB-2837	LAML	WT	WT	WT	WT	WT	WT	WT	WT	WT
TCGA-AB-2978	LAML	WT	WT	WT	WT	WT	WT	WT	WT	WT
TCGA-AB-2879	LAML	WT	WT	WT	WT	WT	WT	WT	WT	WT
TCGA-AB-2931	LAML	WT	WT	WT	WT	WT	WT	WT	WT	WT
TCGA-AB-2932	LAML	WT	WT	WT	WT	WT	WT	WT	WT	WT
TCGA-AB-2844	LAML	WT	WT	WT	WT	WT	WT	WT	WT	WT

TCGA-AB-2872	LAML	WT	WT	WT	WT	WT	WT	WT	WT	WT
TCGA-AB-2839	LAML	WT	WT	WT	WT	WT	WT	WT	WT	WT
TCGA-AB-2948	LAML	WT	WT	WT	WT	WT	WT	WT	WT	WT
TCGA-AB-3011	LAML	WT	WT	WT	WT	WT	WT	WT	WT	WT
TCGA-AB-2956	LAML	WT	WT	WT	WT	WT	WT	WT	WT	WT
TCGA-AB-2817	LAML	WT	WT	WT	WT	WT	WT	WT	WT	WT
TCGA-AB-2806	LAML	WT	WT	WT	WT	WT	WT	WT	WT	WT
TCGA-AB-2823	LAML	WT	WT	WT	WT	WT	WT	WT	WT	WT
TCGA-AB-2973	LAML	WT	WT	WT	WT	WT	WT	WT	WT	WT
TCGA-AB-2930	LAML	WT	WT	WT	WT	WT	WT	WT	WT	WT
TCGA-AB-2896	LAML	WT	WT	WT	WT	WT	WT	WT	WT	WT
TCGA-AB-2895	LAML	WT	WT	WT	WT	WT	WT	WT	WT	WT
TCGA-AB-2942	LAML	WT	WT	WT	WT	WT	WT	WT	WT	WT
TCGA-AB-2928	LAML	WT	WT	WT	WT	WT	WT	WT	WT	WT
TCGA-AB-2833	LAML	WT	WT	WT	WT	WT	WT	WT	WT	WT
TCGA-AB-2983	LAML	WT	WT	WT	WT	WT	WT	WT	WT	WT
TCGA-AB-2835	LAML	WT	WT	WT	WT	WT	WT	WT	WT	WT
TCGA-AB-2836	LAML	WT	WT	WT	WT	WT	WT	WT	WT	WT
TCGA-AB-2841	LAML	WT	WT	WT	WT	WT	WT	WT	WT	WT
TCGA-AB-2842	LAML	WT	WT	WT	WT	WT	WT	WT	WT	WT
TCGA-AB-2870	LAML	WT	WT	WT	WT	WT	WT	WT	WT	WT
TCGA-AB-2936	LAML	WT	WT	WT	WT	WT	WT	WT	WT	WT
TCGA-AB-3007	LAML	WT	WT	WT	WT	WT	WT	WT	WT	WT
TCGA-AB-3001	LAML	WT	WT	WT	WT	WT	WT	WT	WT	WT
TCGA-AB-3000	LAML	WT	WT	WT	WT	WT	WT	WT	WT	WT
TCGA-AB-2941	LAML	WT	WT	WT	WT	WT	WT	WT	WT	WT
TCGA-AB-2939	LAML	WT	WT	WT	WT	WT	WT	WT	WT	WT
TCGA-AB-2815	LAML	WT	WT	WT	WT	WT	WT	WT	WT	WT
TCGA-AB-2840	LAML	WT	WT	WT	WT	WT	WT	WT	WT	WT
TCGA-AB-2807	LAML	WT	WT	WT	WT	WT	WT	WT	WT	WT
TCGA-AB-2858	LAML	WT	WT	WT	WT	WT	WT	WT	WT	WT
TCGA-AB-2848	LAML	WT	WT	WT	WT	WT	WT	WT	WT	WT
TCGA-AB-2940	LAML	WT	WT	WT	WT	WT	WT	WT	WT	WT
TCGA-AB-2988	LAML	WT	WT	WT	WT	WT	WT	WT	WT	WT
TCGA-AB-3008	LAML	WT	WT	WT	WT	WT	WT	WT	WT	WT
TCGA-AB-2998	LAML	WT	WT	WT	WT	WT	WT	WT	WT	WT
TCGA-AB-2813	LAML	WT	WT	WT	WT	WT	WT	WT	WT	WT
TCGA-AB-2884	LAML	WT	WT	WT	WT	WT	WT	WT	WT	WT
TCGA-AB-2918	LAML	WT	WT	WT	WT	WT	WT	WT	WT	WT

TCGA-AB-2846	LAML	WT	WT	WT	WT	WT	WT	WT	WT	WT
TCGA-AB-2925	LAML	WT	WT	WT	WT	WT	WT	WT	WT	WT
TCGA-AB-2818	LAML	WT	WT	WT	WT	WT	WT	WT	WT	WT
TCGA-AB-2959	LAML	WT	WT	WT	WT	WT	WT	WT	WT	WT
TCGA-AB-2938	LAML	WT	WT	WT	WT	WT	WT	WT	WT	WT
TCGA-AB-2849	LAML	WT	WT	WT	WT	WT	WT	WT	WT	WT
TCGA-AB-2917	LAML	WT	WT	WT	WT	WT	WT	WT	WT	WT
TCGA-AB-2914	LAML	WT	WT	WT	WT	WT	WT	WT	WT	WT
TCGA-AB-2810	LAML	WT	WT	WT	WT	WT	WT	WT	WT	WT
TCGA-AB-2853	LAML	WT	WT	WT	WT	WT	WT	WT	WT	WT
TCGA-AB-2995	LAML	WT	WT	WT	WT	WT	WT	WT	WT	WT
TCGA-AB-2933	LAML	WT	WT	WT	WT	WT	WT	WT	WT	WT
TCGA-AB-2834	LAML	WT	WT	WT	WT	WT	WT	WT	WT	WT
TCGA-AB-2820	LAML	WT	WT	WT	WT	WT	WT	WT	WT	WT
TCGA-AB-2909	LAML	WT	WT	WT	WT	WT	WT	WT	WT	WT
TCGA-AB-2898	LAML	WT	WT	WT	WT	WT	WT	WT	WT	WT
TCGA-AB-2992	LAML	WT	WT	WT	WT	WT	WT	WT	WT	WT
TCGA-AB-2868	LAML	WT	WT	WT	WT	WT	WT	WT	WT	WT
TCGA-AB-2934	LAML	WT	WT	WT	WT	WT	WT	WT	WT	WT
TCGA-AB-2855	LAML	WT	WT	WT	WT	WT	WT	WT	WT	WT
TCGA-AB-2949	LAML	WT	WT	WT	WT	WT	WT	WT	WT	WT
TCGA-AB-2987	LAML	WT	WT	WT	WT	WT	WT	WT	WT	WT
TCGA-AB-2980	LAML	WT	WT	WT	WT	WT	WT	WT	WT	WT
TCGA-AB-2991	LAML	WT	WT	WT	WT	WT	WT	WT	WT	WT
TCGA-AB-2967	LAML	WT	WT	WT	WT	WT	WT	WT	WT	WT
TCGA-AB-2913	LAML	WT	WT	WT	WT	WT	WT	WT	WT	WT
TCGA-AB-2867	LAML	WT	WT	WT	WT	WT	WT	WT	WT	WT
TCGA-AB-2865	LAML	WT	WT	WT	WT	WT	WT	WT	WT	WT
TCGA-AB-2955	LAML	WT	WT	WT	WT	WT	WT	WT	WT	WT
TCGA-AB-2891	LAML	WT	WT	WT	WT	WT	WT	WT	WT	WT
TCGA-AB-2943	LAML	WT	WT	WT	WT	WT	WT	WT	WT	WT
TCGA-AB-2822	LAML	WT	WT	WT	WT	WT	WT	WT	WT	WT
TCGA-AB-2966	LAML	WT	WT	WT	WT	WT	WT	WT	WT	WT
TCGA-AB-2946	LAML	WT	WT	WT	WT	WT	WT	WT	WT	WT
TCGA-AB-2979	LAML	WT	WT	WT	WT	WT	WT	WT	WT	WT
TCGA-AB-2874	LAML	WT	WT	WT	WT	WT	WT	WT	WT	WT
TCGA-AB-2877	LAML	WT	WT	WT	WT	WT	WT	WT	WT	WT
TCGA-AB-2808	LAML	WT	WT	WT	WT	WT	WT	WT	WT	WT
TCGA-AB-2900	LAML	WT	WT	WT	WT	WT	WT	WT	WT	WT

TCGA-AB-2903	LAML	WT	WT	WT	WT	WT	WT	WT	WT	WT
TCGA-AB-2984	LAML	WT	WT	WT	WT	WT	WT	WT	WT	WT
TCGA-AB-2999	LAML	WT	WT	WT	WT	WT	WT	WT	WT	WT
TCGA-AB-3012	LAML	WT	WT	WT	WT	WT	WT	WT	WT	WT
TCGA-AB-2863	LAML	WT	WT	WT	WT	WT	WT	WT	WT	WT
TCGA-AB-2965	LAML	WT	WT	WT	WT	WT	WT	WT	WT	WT
TCGA-AB-2971	LAML	WT	WT	WT	WT	WT	WT	WT	WT	WT
TCGA-AB-2972	LAML	WT	WT	WT	WT	WT	WT	WT	WT	WT
TCGA-AB-2875	LAML	WT	WT	WT	WT	WT	WT	WT	WT	WT
TCGA-AB-2993	LAML	WT	WT	WT	WT	WT	WT	WT	WT	WT
TCGA-AB-2990	LAML	WT	WT	WT	WT	WT	WT	WT	WT	WT
TCGA-AB-2825	LAML	WT	WT	WT	WT	WT	WT	WT	WT	WT
TCGA-AB-2880	LAML	WT	WT	WT	WT	WT	WT	WT	WT	WT
TCGA-AB-2964	LAML	WT	WT	WT	WT	WT	WT	WT	WT	WT
TCGA-AB-2919	LAML	WT	WT	WT	WT	WT	WT	WT	WT	WT
TCGA-AB-3009	LAML	WT	WT	WT	WT	WT	WT	WT	WT	WT
TCGA-AB-2963	LAML	WT	WT	WT	WT	WT	WT	WT	WT	WT
TCGA-AB-2921	LAML	WT	WT	WT	WT	WT	WT	WT	WT	WT
TCGA-AB-2857	LAML	WT	WT	WT	WT	WT	WT	WT	WT	WT
TCGA-AB-2935	LAML	WT	WT	WT	WT	WT	WT	WT	WT	WT
TCGA-AB-2873	LAML	WT	WT	WT	WT	WT	WT	WT	WT	WT
TCGA-AB-2924	LAML	WT	WT	WT	WT	WT	WT	WT	WT	WT
TCGA-AB-2838	LAML	WT	WT	WT	WT	WT	WT	WT	WT	WT
TCGA-AB-3005	LAML	WT	WT	WT	WT	WT	WT	WT	WT	WT
TCGA-AB-3002	LAML	WT	WT	WT	WT	WT	WT	WT	WT	WT
TCGA-AB-2826	LAML	WT	WT	WT	WT	WT	WT	WT	WT	WT
TCGA-AB-2911	LAML	WT	WT	WT	WT	WT	WT	WT	WT	WT
TCGA-AB-2862	LAML	WT	WT	WT	WT	WT	WT	WT	WT	WT
TCGA-AB-2975	LAML	WT	WT	WT	WT	WT	WT	WT	WT	WT
TCGA-AB-2910	LAML	WT	WT	WT	WT	WT	WT	WT	WT	WT
TCGA-AB-2888	LAML	WT	WT	WT	WT	WT	WT	WT	WT	WT
TCGA-AB-2970	LAML	WT	WT	WT	WT	WT	WT	WT	WT	WT
TCGA-AB-2803	LAML	WT	WT	WT	WT	WT	WT	WT	WT	WT
TCGA-AB-2832	LAML	WT	WT	WT	WT	WT	WT	WT	WT	WT
TCGA-AB-2814	LAML	WT	WT	WT	WT	WT	WT	WT	WT	WT
TCGA-AB-2869	LAML	WT	WT	WT	WT	WT	WT	WT	WT	WT
TCGA-AB-2944	LAML	WT	WT	WT	WT	WT	WT	WT	WT	WT
TCGA-AB-2950	LAML	WT	WT	WT	WT	WT	WT	WT	WT	WT
TCGA-AB-2927	LAML	WT	WT	WT	WT	WT	WT	WT	WT	WT

TCGA-AB-2916	LAML	WT	WT	WT	WT	WT	WT	WT	WT	WT
TCGA-AB-2889	LAML	WT	WT	WT	WT	WT	WT	WT	WT	WT
TCGA-AB-2901	LAML	WT	WT	WT	WT	WT	WT	WT	WT	WT
TCGA-AB-2845	LAML	WT	WT	WT	WT	WT	WT	WT	WT	WT
TCGA-AB-2969	LAML	WT	WT	WT	WT	WT	WT	WT	WT	WT
TCGA-AB-2854	LAML	WT	WT	WT	WT	WT	WT	WT	WT	WT
TCGA-AB-3006	LAML	WT	WT	WT	WT	WT	WT	WT	WT	WT
TCGA-AB-2887	LAML	WT	WT	WT	WT	WT	WT	WT	WT	WT
TCGA-AB-2805	LAML	WT	WT	WT	WT	WT	WT	WT	WT	WT
TCGA-AB-2860	LAML	WT	WT	WT	WT	WT	WT	WT	WT	WT
TCGA-AB-2986	LAML	WT	WT	WT	WT	WT	WT	WT	WT	WT
TCGA-AB-2812	LAML	WT	WT	WT	WT	WT	WT	WT	WT	WT
TCGA-AB-2811	LAML	WT	WT	WT	WT	WT	WT	WT	WT	WT
TCGA-AB-2915	LAML	WT	WT	WT	WT	WT	WT	WT	WT	WT
TCGA-AB-2881	LAML	WT	WT	WT	WT	WT	WT	WT	WT	WT
TCGA-AB-2890	LAML	WT	WT	WT	WT	WT	WT	WT	WT	WT
TCGA-AB-2981	LAML	WT	WT	WT	WT	WT	WT	WT	WT	WT
TCGA-AB-2830	LAML	WT	WT	WT	WT	WT	WT	WT	WT	WT
TCGA-AB-2954	LAML	WT	WT	WT	WT	WT	WT	WT	WT	WT
TCGA-AB-2952	LAML	WT	WT	WT	WT	WT	WT	WT	WT	WT

Table S2: Splicing factors reported to be significantly altered in previous studies where *U2AF1* is somatically mutated.

**Splicing Factor**	**Reference**
HNRNPK	[2]
PRPF40B	[3]
RBM10	[4]
SF1	[3]
SF3A1	[3]
SF3B1	[3]
SRSF2	[3]
U2AF2	[3]
ZRSR2	[3]

Table S3: Mitotic cell cycle genes that show differential splicing in the presence of the *U2AF1* S34F/Y mutation.

**Gene**	**GO:0000278 :mitotic cell cycle**	**GO:0000087 :M phase of mitotitic cell cycle**	**U2AF1 S34F/Y-associated differential splicing in AML**	**U2AF1 S34F-associated differential splicing in lung adenocarci-noma**
ANAPC1	x	x	x	
ANAPC10	x	x	x	
ANAPC5	x	x	x	
ANLN	x		x	
BLZF1	x	x		x
BUB3	x	x	x	
CCNH	x		x	
CDC27	x	x	x	
CENPO	x	x	x	
CEP192	x		x	
CHEK2	x		x	
CKAP5	x	x	x	
DDX11	x		x	
EZH2	x		x	
FAM111A	x	x	x	
FGFR1OP	x		x	
ITGB3BP	x	x	x	
LIG1	x		x	
MNAT1	x		x	
NAE1	x			x
NUSAP1	x		x	
OPTN	x			x
PCM1	x		x	
RBL1	x		x	
SEC13	x	x	x	
SFI1	x		x	
SH2B1	x		x	
TERF1	x		x	
TFDP1	x		x	
TFDP2	x		x	
WAPAL	x	x	x	

Table S4: Cancer Gene Census genes differentially spliced in the presence of a *U2AF1* S34F/Y mutation.

Gene	U2AF1 S34F/Y-associated differential splicing in AML	U2AF1 S34F-associated differential splicing in lung adenocarcinoma
ATRX	x	
BCL9		x
BCOR	x	
CHCHD7	x	x
CHEK2	x	
CTNNB1	x	x
EIF4A2	x	
EZH2	x	
FGFR1OP	x	
FIP1L1	x	
FLT3	x	
KDM6A	x	
NCOA2	x	
NIN	x	
PCM1	x	
PDGFRA	x	
PICALM	x	x
RAD51L1		x
RALGDS	x	
U2AF1		x
WHSC1	x	

Table S5: Taqman assays (Life Technologies) used for quantitative RT-PCR of splicing events in *CHCHD7, CHEK2, CTNNB1, KARS,* and, *PTBP1*.

Gene	Target	AssayID
CHCHD7	constitutive	Hs00225599_m1
CHCHD7	inclusion isoform	Hs03044402_m1
CHEK2	constitutive	Hs01007281_m1
CHEK2	inclusion isoform	custom
CTNNB1	constitutive	Hs00991811_m1
CTNNB1	inclusion isoform	Hs00991812_g1
KARS	constitutive	Hs00271471_m1
KARS	inclusion isoform	custom
PTBP1	constitutive	Hs00738538_g1
PTBP1	inclusion isoform	Hs00914697_g1

References

1. Przychodzen B, Jerez A, Guinta K, Sekeres MA, Padgett R, et al. (2013) Patterns of missplicing due to somatic U2AF1 mutations in myeloid neoplasms. Blood. doi:10.1182/blood-2013-01-480970.

2. The Cancer Genome Atlas Research Network (2013) Genomic and Epigenomic Landscapes of Adult De Novo Acute Myeloid Leukemia. N Engl J Med. doi:10.1056/NEJMoa1301689.

3. Yoshida K, Sanada M, Shiraishi Y, Nowak D, Nagata Y, et al. (2011) Frequent pathway mutations of splicing machinery in myelodysplasia. Nature 478: 64–69. doi:10.1038/nature10496.

4. Imielinski M, Berger AH, Hammerman PS, Hernandez B, Pugh TJ, et al. (2012) Mapping the hallmarks of lung adenocarcinoma with massively parallel sequencing. Cell 150: 1107–1120. doi:10.1016/j.cell.2012.08.029.
